# Peptidoglycan derived from *Lacticaseibacillus rhamnosus* and *Lactobacillus acidophilus* suppress TLR2/1-mediated inflammation in bovine endometrial epithelial cells

**DOI:** 10.3389/fimmu.2025.1622307

**Published:** 2025-06-18

**Authors:** Elham Waehama, Kenji Fukuda, Alireza Mansouri, Malinda Hulugalla, Ihshan Akthar, Mohamed Samy Yousef, Akio Miyamoto

**Affiliations:** ^1^ Global Agromedicine Research Center (GAMRC), Obihiro University of Agriculture and Veterinary Medicine, Obihiro, Japan; ^2^ Faculty of Agriculture, Princess of Naradhiwas University, Narathiwat, Thailand; ^3^ Department of Theriogenology, Faculty of Veterinary Medicine, Assiut University, Assiut, Egypt

**Keywords:** peptidoglycan, *Lactobacillus* species, *Staphylococcus aureus*, PAM3, inflammation, bovine endometrial epithelial cells

## Abstract

Bacteria and associated products are factors in the pathogenesis of bovine endometrial inflammation, contributing to reproductive dysfunction. While peptidoglycan derived from *Staphylococcus aureus* (PGN-Sa) has been demonstrated to induce pro-inflammatory responses and disrupt sperm–immune interactions in bovine endometrial epithelial cells (BEECs) via Toll-like receptor 2/1 (TLR2/1), the immunomodulatory potential of peptidoglycan from lactic acid bacteria (LAB) within the female reproductive tract remains unexplored. This study investigated the *in vitro* immunomodulatory effects of LAB-derived peptidoglycan (PGN-L) on TLR2/1-mediated inflammation in BEECs, with the specific TLR2/1 agonist PAM3CSK4 (PAM3) as an inflammatory stimulus. PGN-L was extracted and characterized from *Lacticaseibacillus rhamnosus* (PGN-Lr) and *Lactobacillus acidophilus* (PGN-La), and its structural composition was compared to that of commercial PGN-Sa. Subsequently, BEECs were pre-incubated with PGN-L (Lr, La) or PGN-Sa (1 ng/mL) for 24 h before stimulation with PAM3 (100 ng/mL) for 3 h. The expression of inflammatory genes (*TNF*, *CXCL8*, *IL1B*, and *PTGES*) and TLRs (*TLR1*, *TLR2*, *TLR4*, and *TLR6*) was quantified by RT-qPCR. The protein expression of TNF, PTGES, and TLR2 was detected using immunofluorescence, while PGE_2_ concentrations in the culture media were measured by ELISA. PGN-Lr and PGN-La shared the GlcNAc-MurNAc backbone with PGN-Sa, while PGN-L had a unique modification. PGN-L and PGN-Sa contained lysine at the cross-bridge stem, composed of glycine in PGN-Sa and likely modified D-aspartate in PGN-L. While PGN-Sa and PAM3 significantly upregulated the expression of inflammatory mediators, neither PGN-Lr nor PGN-La alone induced a pro-inflammatory response in BEECs. Importantly, pretreatment with both PGN-Lr and PGN-La significantly reduced PAM3-induced inflammatory gene expression and reduced PGE_2_ secretion. *In silico* molecular findings suggested a potential mechanism whereby PGN-L may act as a TLR2/1 antagonist, contrasting with the agonistic effects of PGN-Sa and PAM3, which promoted TLR2/1 heterodimerization. These findings suggest that PGN-Lr and PGN-La can suppress TLR2/1-mediated uterine inflammation *in vitro*, by potentially modulating TLR2/1 signaling in BEECs. Further investigation of PGN-L holds promise for the development of therapeutic strategies to enhance bovine reproductive efficiency.

## Introduction

The bovine reproductive tract hosts a complex and dynamic microbial ecosystem, encompassing both Gram-negative and Gram-positive bacteria that exert significant influence on uterine health and disease. Certain Gram-negative bacteria, such as *Escherichia coli*, and Gram-positive bacteria, including *Trueperella pyogenes*, are well-established etiological agents of postpartum uterine infections like metritis and endometritis, leading to substantial declines in fertility and productivity in dairy cattle (1, 2). On the other hand, *Staphylococcus* and *Streptococcus* species, are also commonly isolated and may contribute to both commensal balance and pathogenic disruptions depending on host immunity and environmental factors (3, 4). In addition, lactic acid bacteria (LAB), a particularly dominant *Lactobacillus* species of a healthy female reproductive tract microbiota, play a crucial role in maintaining mucosal homeostasis and protecting against pathogenic infections in humans (5). They also constitute a significant component of the healthy bovine uterine microbiota, which is involved in increasing the conception rate (6), and are recognized for their contribution to mucosal defense through the production of antimicrobial compounds and the maintenance of an acidic environment (7). Their presence has been associated with improved uterine health and reduced incidence of pathogenic bacterial colonization in the postpartum period (8, 9).

The cell wall components of major uterine pathogens, lipopolysaccharide (LPS) from Gram-negative bacteria and peptidoglycan (PGN) from Gram-positive bacteria, play critical roles in initiating uterine inflammation. Specifically, *E. coli*-derived LPS primarily engages toll-like receptor 4 (TLR4), while *Staphylococcus aureus*-derived peptidoglycan (PGN-Sa) is predominantly recognized by TLR2 (10–12). Activation of these TLRs triggers downstream signaling pathways, notably the nuclear factor kappa-B (NF- κB) pathway, ending in the production of pro-inflammatory cytokines such as interleukin-1 beta (IL1B) and tumor necrosis factor (TNF), which contribute to endometrial tissue damage and impaired fertility (13, 14). Interestingly, peptidoglycan derived from lactic acid bacteria (PGN-L), particularly from *Lactobacillus* species, has demonstrated anti-inflammatory properties in other biological systems (15, 16), suggesting a potential to modulate TLR signaling and suppress excessive cytokine production (16).

In the bovine endometrium, both LPS and PGN, representing Gram-negative and Gram-positive bacteria, respectively, are key ligands for the TLR family, particularly TLR4 and TLR2, thereby influencing the intricate immune network within bovine endometrial epithelial cells (BEECs) (11). Under physiological conditions, TLR signaling plays a vital role in maintaining immune surveillance and mucosal homeostasis through the controlled production of cytokines and the preservation of tissue integrity (17).

It is noteworthy that the sperm in the uterus triggers a transient pro-inflammatory response (TNF, IL1B, CXCL8, and PGES) considered essential for the clearance of excess sperm and the establishment of an optimal uterine environment for subsequent embryo implantation (18, 19). However, in pathological conditions such as postpartum endometritis, excessive or dysregulated activation of TLRs by LPS and PGN leads to overproduction of pro-inflammatory mediators, disruption of the epithelial barrier, and recruitment of immune cells, ultimately contributing to endometrial damage and impaired fertility in cattle (20). This dual role of TLR-mediated signaling highlights the delicate balance between protective immunity and harmful inflammation within the bovine reproductive tract, emphasizing the need for regulated immune responses to microbial components (10).

Previous research has indicated that PGN-Sa can inhibit the production of key steroid hormones, progesterone (P4), and androstenedione (A4) in bovine theca cells (21) while also inducing inflammatory responses through TLR2 signaling. Notably, PGN-Sa has also been shown to suppress the physiological sperm-induced inflammatory response in BEECs (11), highlighting its immunomodulatory capacity within the reproductive tract. In contrast, the impact of PGN-L on the bovine reproductive system remains largely unexplored. Therefore, this study aimed to investigate the potential immunomodulatory effects of PGN-L, especially derived from *Lacticaseibacillus rhamnosus* (PGN-Lr) and *Lactobacillus acidophilus* (PGN-La), in comparison to PGN-Sa, on inflammation in BEECs, particularly focusing on inflammation mediation through the TLR2/1 signaling pathway activated by PAM3CSK4 (PAM3) as a specific agonist. This research represents a fundamental step toward understanding the role of bacterial components in uterine immune regulation and overall reproductive health.

## Methods

### Experimental design

**Figure 1:**
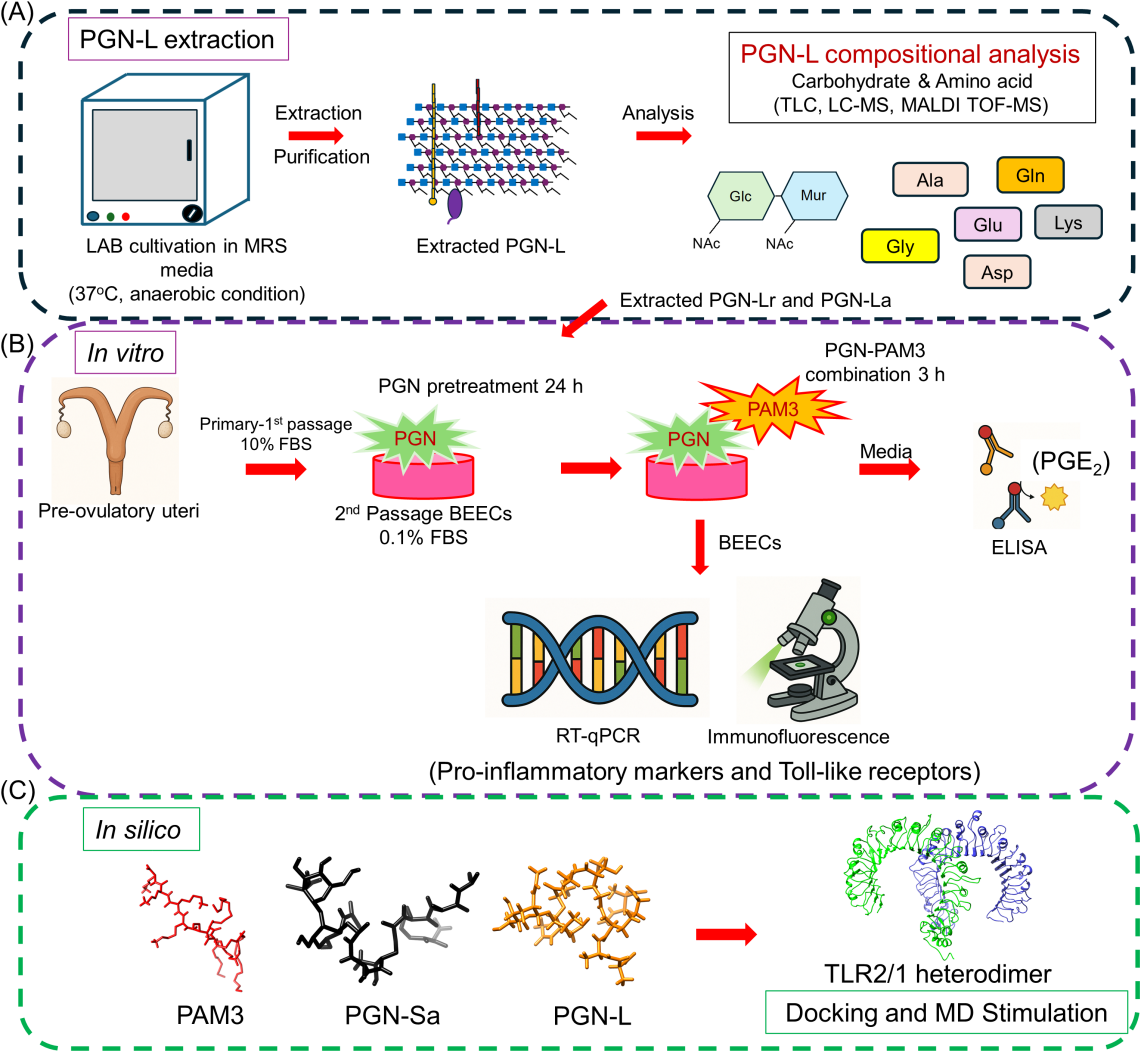
Schematic illustration of the experimental methodology. **(A)** Extraction of the LAB-derived peptidoglycans (PGN-L). The LAB was cultivated and extracted. After that, the purification of PGN-L was conducted. Following the extracted PGN-L, the chemical composition has been further analyzed. **(B)** A simplified *in vitro* co-culture model was used to investigate the inflammatory response of BEECs. BEECs were pretreated with PGN-(Lr, La, Sa) for 24 h and, after that, stimulated PAM3 for 3 h. The RT-qPCR and immunostaining analyses were conducted to investigate the mRNA and protein expression of pro-inflammatory markers and Toll-like receptors (TLRs). ELISA was used to measure the pro-inflammatory lipid mediator concentration of PGE_2_ in co-cultured media. **(C)** *In silico* analysis. Docking and molecular dynamics (MD) simulation was used to investigate the immune impact of potential ligands within the PGN-Sa and PGN-L. The binding affinity for TLR2 dimers of two significant components of PGN-Sa and PGN-L was explored.

Initially, PGN-Lr and PGN-La were extracted, and biochemical analysis was conducted to analyze and compare their structural composition to that of commercially sourced PGN-Sa. Then, *in vitro* investigations were conducted to investigate the impact of PGN on LPS/PAM3-induced inflammation in the bovine uterus at the cellular level. Briefly, BEECs were pretreated with PGN (Lr, La, Sa) for 24 h and stimulated with LPS for 1 h (16) or PAM3 for 3 h (11, 22). Pro-inflammatory gene and protein expression were investigated via RT-qPCR and immunofluorescence (IF) analysis in the BEECs, and pro-inflammatory mediator (PGE_2_) concentration in conditioned media was evaluated by using commercially available ELISA kit. *In silico* analysis was conducted to investigate the immune impact of potential ligands (PGN) on TLR2/1 dimerization. The detailed experimental methodology is shown in **Figure 1**.

### Pathogen-derived TLR2/4 ligands

PGN from Gram-positive *Staphylococcus aureus* (Sigma-Aldrich, MFCD00212486, USA) (PGN-Sa) and the extracted PGN from Gram-positive *Lacticaseibacillus rhamnosus* ATCC 53103 (PGN-Lr) and *Lactobacillus acidophilus* ATCC 4356 (PGN-La) were used as natural TLR2 ligands, respectively, for treatments (11). PAM3 (PAM3CSK4: Pam3CysSerLys4), synthetic triacylated lipopeptide, TLR2/TLR1 agonist (InvivoGen - ab142085, Abcam, USA) (22), and LPS (Gram-negative *Escherichia coli* O55: B5, Sigma-Aldrich, USA) were used to induce inflammation in BEECs, and TLR2/1 antagonist (CU-CPT22; 614305, Sigma-Aldrich) was used to block the TLR2/1 pathway.

### Preparation of PGN from *Lactobacillus* strains

PGN-L samples were purified from *Lacticaseibacillus rhamnosus* ATCC53103 and *Lactobacillus acidophilus* ATCC 4356 according to a previously described method with modification (16, 23). Briefly, bacterial cells were harvested from 1 L of de Man–Rogosa–Sharpe broth (Oxoid) statically and anaerobically cultivated at 37°C for 24 h. The cells were washed three times with phosphate-buffered saline, suspended in 4% SDS (1 g of bacterial cells per milliliter of 4% SDS), and boiled for 30 min. After cooling, the cell pellet was collected by centrifugation (10,000 × *g*, 4°C, 10 min) and thoroughly washed with distilled water. The cell pellet was sonicated at 1,420 J on ice for 10 min (three times); then, cell debris was collected by centrifugation (27,000 × *g*, 4°C, 1 h) and thoroughly washed with distilled water. The pellet was suspended in 10 mL of 100 mM Tris-HCl (pH 7.5) containing 1 mg/mL DNase I (Sigma-Aldrich), 5 mg/mL RNase A (Sigma-Aldrich), and 20 mM MgSO_4_ and then incubated at 37°C for 2 h. Moreover, 10 µg/mL trypsin (Sigma-Aldrich) and 10 mM CaCl_2_ were further added to the suspension, which was then incubated at 37°C for 14 h. Cell debris was collected by centrifugation (27,000 × *g*, 4°C, 1 h), and 10 mL of 1% SDS was added to the pellet, and then this was boiled for 10 min. The cell debris was collected and washed as described above. Next, 50 mL of 10% trichloroacetic acid was added to the pellet, the mixture was incubated at 4°C for 24 h, and cell debris was collected and washed as described above. Then, 30 mL of absolute methanol was added to the cell debris, mixed thoroughly, and then filtered using a glass filter. The cell debris on the glass filter was collected and suspended in 30 mL of 1:1 mixture of absolute methanol and chloroform. The suspension was filtered by using a glass filter, collected, and suspended in 10 mL of 100 mM Tris-HCl (pH 7.5) containing 1 mg/mL pepsin (Sigma-Aldrich) and 2 mg/mL protease from *A. saitoi* (Sigma-Aldrich). The suspension was incubated at 37°C for 24 h. The debris was collected by centrifugation (27,000 × *g*, 4°C, 1 h). Then, 15 mL of 1% SDS was added. After boiling for 10 min, cell debris was collected and washed as detailed above. Then, 20 mL of 5 mM sulfuric acid was added to the debris, incubated at 90°C for 5 min, and cooled down on ice. The cell debris was collected and washed as described above and then dialyzed against 1 L of distilled water by using 1,000-kDa cutoff dialysis membrane (Repligen) for 5 days with the distilled water being changed once per day. After that, the retentate was collected as purified PGN, lyophilized, and stored at room temperature (RT) in a desiccator.

### PGN composition analysis

To assess the purity of purified PGN, the carbohydrate and amino acid composition of PGN was determined by thin-layer chromatography (TLC), LC-MS, and amino acid analysis, respectively. PGN-Sa was used as the reference substance. First, PGN (1 mg each) was thoroughly hydrolyzed in 1 M or 6 M HCl at 105°C for 16 h. Each 10 µL of the hydrolysates was loaded onto a silica gel 60 plate (Merck) and developed with *n*-butanol/ethanol/water (2:1:1, v/v) at RT. Each 5 µg of *N*-acetylglucosamine (GlcNAc, Sigma-Aldrich) and *N*-acetylmuraminic acid (MurNAc, Sigma-Aldrich) was used as standard. The carbohydrates were visualized by spraying 5% sulfuric acid in ethanol and heating. In addition, 1 µL of each PGN was hydrolyzed with 6 M HCl and then subjected to separation on Shodex VG-50 2D (2.0 × 150 mm) connected to Agilent InfinityLab LC/MSD system (single quadrupole, Agilent Technologies) at 60°C at a flow rate of 0.15 mL/min. Elution was performed with 88%–50% linear gradient of acetonitrile in 0.05% ammonia. The MS parameters were as follows: ESI-negative mode, dry gas flow rate at 10 L/min, dry gas temperature at 250°C, nebulizer pressure at 60 psi, capillary voltage at 3,500 V, and fragmentor voltage at 40 V. Next, PGN (1 mg each) was thoroughly hydrolyzed in 6 M HCl at 105°C for 16 h. The amino acid composition of the hydrolysates was analyzed using LA8080 AminoSAAYA (Hitachi HighTech) according to the manufacturer’s instructions.

### Uterine sampling

Healthy bovine uteri from post-pubertal, nonpregnant cows were carefully examined macroscopically and confirmed to be free of inflammation, abnormal appearances such as color, odor, or any pathological lesions, and then ipsilateral uterine horns were collected from the slaughterhouse (Doto Plant Tockachi Factory, Obihiro, Japan). The reproductive tract was kept at 4°C and transported to the laboratory under sterilized conditions for cell culture (24).

### BEEC culture

Isolation and culture of epithelial cells were conducted following the method previously described (25) with modifications. The uterine lumen was washed twice with 25 mL of sterile Ca^2+^ and Mg^2+^ free Hanks’ balanced salt solution (HBSS) supplemented with 1% v/v penicillin–streptomycin (15140122, Gibco, Thermo Fisher Scientific, Waltham, MA, USA) and 1 mg/mL bovine serum albumin (BSA) (A7888, Sigma-Aldrich, St. Louis, MO, USA). An enzyme solution (25 mL) (sterile HBSS containing 500 μg/mL collagenase I [C2674, Sigma-Aldrich], 50 μg/mL deoxyribonuclease I [043-26773, FUJIFILM Wako Pure Chemical Corporation, Osaka, Japan], and 1 mg/mL BSA) was then infused into the uterine lumen through a sterile vinyl catheter. Epithelial cells were isolated by incubation at 38.5°C for 45 min. The cell suspension obtained was then filtered through a double layer of nylon mesh (100 μm). The filtrate was washed twice by centrifugation (10 min at 180 × *g*) with Dulbecco’s modified Eagle’s medium (DMEM/F12, 12400024, Gibco, Thermo Fisher Scientific) supplemented with 1% v/v amphotericin B (15290018, Gibco, Thermo Fisher Scientific), 1% v/v penicillin–streptomycin, and 100 μg/mL BSA. The final pellet was resuspended in equilibrated 5 mL of culture media of DMEM/F12 supplemented with 1% v/v amphotericin B, 50 μg/mL gentamicin sulfate (G1264, Sigma-Aldrich), and 10% v/v heat-inactivated fetal bovine serum (FBS) (S1400, Ireland-origin, Biowest, Nuaillé, France). The cells were then seeded in 25-cm^2^ flasks (156340, Nunc, Thermo Fisher Scientific) at 38.5°C in a humidified atmosphere of air with 5% CO_2_. The culture medium was replaced with new media every 48 h. Upon reaching 70%–80% confluence, the cells were collected by trypsinization [2.5 mg/mL trypsin (7409, Sigma-Aldrich) in 0.2 mg/mL EDTA (D05125 Sigma-Aldrich)] and then transferred in 25-cm^2^ flasks (156340, Nunc, Thermo Fisher Scientific) at 38.5°C in a humidified atmosphere of air with 5% CO_2_ as the secondary cell culture similar to the primary cell culture. The culture medium was replaced with new media every 48 h. Upon reaching 70%–80% confluence, the cells were again collected by trypsinization [2.5 mg/mL trypsin (7409, Sigma-Aldrich) in 0.2 mg/mL EDTA (D05125 Sigma-Aldrich)] and transferred in 24-well plates (142475, Nunc, Thermo Fisher Scientific) and cultured up to around 90% confluence (tertiary cell culture). β-estradiol (E8875, Sigma-Aldrich,1 ng/mL) and progesterone (P8783, Sigma-Aldrich, 50 pg/mL) were added at preovulatory concentrations (25) in the cell culture media (DMEM/F12, 1% v/v amphotericin B, 50 μg/mL gentamicin, and 5% v/v FBS) (24). The purity of BEECs was determined and confirmed through cytokeratin immunostaining and their characteristic epithelial morphology. The purity of cultured BEECs was >98% (26).

### BEEC stimulation with LPS/PAM3 and PGN

The PAM3 and LPS used in this study were based on our previous experiments, where we observed that 100 ng/mL PAM3 effectively activated TLR2/1 and induced sufficient pro-inflammatory markers (TNF, IL1B, CXCL8, and PGES) across different time points (22). Similarly, 1 ng/mL LPS was sufficient to induce a strong inflammatory response via TLR4 (22). On the one hand, BEECs were pre-incubated with PGN-L (Lr, La) or PGN-Sa (0.01, 0.1, and 1 ng/mL) for 24 h (11) prior to stimulation with PAM3 (100 ng/mL) for 3 h (22, 27). After that, RT-qPCR and immunostaining analyses were conducted to investigate the mRNA and protein expression of inflammatory cytokines and TLRs. ELISA was used to analyze co-cultured media for the inflammatory lipid mediator concentration of PGE_2_. On the other hand, BEECs were pre-incubated with PGN-Lr (5 and 100 μg/mL) or (0.1, 1, and 10 ng/mL) for 24 h prior to stimulation with LPS (1 ng/mL) for 1 h (16, 22). After that, RT-qPCR was conducted to investigate the mRNA expression of key inflammatory markers (*TNF*, *IL1B*, *CXCL8*, and *PGES*) and *TLRs* (27).

### BEEC viability

At the end of the experiment, BEECs were collected using trypsin [2.5 mg/mL trypsin (7409, Sigma-Aldrich) in 0.2 mg/mL EDTA (D05125 Sigma-Aldrich)]. Aliquots of the isolated cell suspension were stained with 0.4% trypan blue (15250061, Gibco, Thermo Fisher Scientific) and counted in a hemocytometer under the microscope (BZ-X800, Keyence). The BEEC viability was classified by counting uncompromised cells (live cells and unstained) and compromised cells (dead cells, both floating and detached cells, and stained blue) (28). Then, the proportion of BEEC viability (% of live cells) was calculated (29).

### RNA extraction, cDNA synthesis, and PCR

RNA extraction, cDNA synthesis, and RT-qPCR followed the previous protocol (30). Total RNA was extracted from endometrial epithelial cells using TRIzol. Briefly, 100 μL of chloroform (038-02606, FUJIFILM Wako Pure Chemical Corporation) was added to each cell lysate and incubated at room temperature (RT) for 5 min. The samples were centrifuged at 12,000 × *g* for 15 min at 4°C. The RNA containing the aqueous layer was carefully transferred to a new microtube, and 250 μL of 100% 2-propanol (164-08335, FUJIFILM Wako Pure Chemical Corporation) was added to the sample and incubated at RT for 10 min. The samples were centrifuged at 12,000 × *g* for 15 min at 4°C, and the supernatant was removed, leaving the RNA pellet. The pellet was washed with 450 μL of 75% ethanol (057-00456, FUJIFILM Wako Pure Chemical Corporation) and centrifuged at 12,000 × *g* for 10 min at 4°C. The extracted RNA was resuspended in RNA storage solution (AM7001, Invitrogen, Thermo Fisher Scientific) and quantified using a spectrophotometer (DS-11, DeNovix, Wilmington, DE, USA).

As previously described, 1 μg of RNA was used for cDNA synthesis (11, 22). RNase-Free DNase kit (M6101, Promega Corporation, Madison, WI, USA) was used during step 1 to remove any residual genomic DNA, and 1 μg of extracted RNA was incubated for 30 min at 37°C in a thermal cycler (Eppendorf, Hamburg, Germany) with the first mixture consisting of 1 μL of RQ1 RNase-free DNase 10X Reaction Buffer, 2 μL of RQ1 RNase-free DNase, and ultrapure DNase/RNase free distilled water (10977015, Invitrogen, Thermo Fisher Scientific) to a final volume of 10 μL. Moreover, 1 μL of the RQ1 DNase stop solution was added for 10 min at 65°C to stop the activity of the remaining DNase enzyme during the second step. The SuperScript^®^ II Reverse Transcriptase kit (18064014, Invitrogen, Thermo Fisher Scientific) synthesized single-strand cDNA. Then, 7 μL of the second mixture containing 1.5 μL of 3 μg/μL random primer (48190011, Invitrogen, Thermo Fisher Scientific), 1.5 μL of 10 mM PCR Nucleotide Mix (dNTP) (11814362001, Roche Diagnostics, Mannheim, Germany), and 4 μL of nuclease-free water was added to the DNase-treated RNA mixture at 65°C for 5 min. Then, the mixture was incubated at 42°C for 2 min with the third mixture of 6 μL of 5X First-Strand Buffer, 3 μL of 0.1 M dithiothreitol, and 1.5 μL of 40 units/μL Ribonuclease Inhibitor Recombinant (SIN-201, Toyobo, Osaka, Japan). Finally, 0.2 μL of 200 units/μL SuperScript™ II Reverse Transcriptase was added to the mixture and incubated at 25°C for 10 min, at 42°C for 50 min, and at 70°C for 15 min. The synthesized cDNA was stored at -30°C until its use for RT-qPCR. The RT-qPCR of target genes, tumor necrosis factor (*TNF*), C-X-C motif chemokine ligand 8 (*CXCL8*), interleukin 1 beta (*IL1B*), prostaglandin E synthase (*PTGES*), Toll-like receptor 1 (*TLR1*), Toll-like receptor 2 (*TLR2*), Toll-like receptor 4 (*TLR4*), Toll-like receptor 6 (*TLR6*), and actin beta (*ACTB*) was carried out by using SYBR Green Supermix (1725271B02, Bio-Rad Laboratories, Hercules, CA, USA) using an iCycler iQ (Bio-Rad Laboratories) (11). A total of 10 µL of the reaction mix containing 2 µL/sample synthesized cDNA, 5 µL of SYBR Green Supermix, with 0.1 µL of the forward and reverse targeted primer pairs (**Supplementary Table S1**), and 2.8 µL ultrapure DNase/RNase free distilled water was used for the amplification program. Firstly, the initial activation step was for 15 min at 95°C, followed by 40 cycles of PCR (15 s of denaturation at 95°C, 15 s of annealing at 55°–58°C, and 30 s of extension at 72°C). The calculated cycle threshold (Ct) values were normalized using *ACTB* as an internal housekeeping gene by applying the ΔΔCt method to quantify the fold change between samples (31).

### Immunofluorescence

The monolayer BEECs were cultured on 24-well plates (13-mm-diameter glass coverslips) with 90% cell growth confluency and then pretreated with PGN (Sa, Lr, La) at 1 ng/mL for 24 h and stimulated PAM3 at 100 ng/mL for 3 h. The cells were washed with PBS twice, fixed with 2 mL of 4% paraformaldehyde for 15 min at room temperature, and then washed twice with PBS. After that, the cells were permeabilized with 2 mL of 0.1% Triton-X10 in PBS for 15 min on ice, and then washed three times with PBS. Afterward, the monolayer cells were blocked by using 2 mL of blocking buffer (5% BSA in PBS) for 1 h at room temperature. The primary antibodies were coated in the cell (TNF, PTGES, and TLR2) for labeling (**Supplementary Table S2**) in a humid chamber at 4°C for 24 h. After washing the cells with PBS for five times and then incubating with Alexa Flour conjugated secondary antibody (**Supplementary Table S2**) for 1 h at 4°C, the cells were washed with PBS six times, and then a VECTASHIELD mounting medium containing DAPI was used (H-1200, Vector Laboratories, CA, USA). Finally, the sections were observed under a fluorescence microscope (BZ-X800, Keyence) to visualize the distribution of TNF (normal mouse IgG replaced the primary antibody in the IgG control), PTGES, and TLR2 (normal rabbit IgG replaced the primary antibody in the IgG control) proteins in the BEECs. The fluorescence intensity of the images was analyzed by using ImageJ (27).

### Quantification of PGE2 levels

At the end of the co-culture period, BEEC-conditioned media were collected, centrifuged twice at 1000 g for 10 min at 4°C, and kept at -80°C for PGE2 quantification. A commercially available PGE2-ELISA kit (Item no.14010, Cayman Chemical, Ann Arbor, MI, USA) was used to determine PGE2 concentrations in BEEC-conditioned media. A 50-µL aliquot of each sample was analyzed according to the manufacturer’s instructions. All samples were run in duplicate. Optical density (OD) readings were performed at 420 nm. The control group was run to determine the baseline concentration of PGE2 in DMEM culture media. This experiment was repeated four times using epithelial cells from three different uteri.

### *In silico* study

The effects of different peptidoglycans (PGN-L and PGN-Sa) on the bovine TLR2/1 receptor were investigated. Based on the chemical composition analysis, we designed our PGN molecules for *in silico* analysis. We performed computational experiments to evaluate the binding affinities of each PGN and their potential to interfere with PAM3-induced TLR2/1 dimerization. For this purpose, a 3D structure of the bovine TLR2/1 dimer was generated based on previously reported models (32, 33). The PDB file of PAM3 (PDB ID: 2Z7X) was obtained, and the structures of PGN-L and PGN-Sa were constructed according to the chemical details illustrated in **Supplementary Figure S1**. The main structural differences between PGN-L and PGN-Sa lie in their peptide and backbone regions (34, 35). Specifically, after the alanine (Ala) residue, PGN-Sa contains D-*iso*-glutamine (Gln), whereas PGN-L contains D-glutamic acid (Glu)-NH_2_. In the backbone, PGN-Sa features a glycine (Gly) residue, while PGN-L includes aspartic acid (Asp)-NH_2_.

In the first phase, molecular docking was performed to obtain initial ligand–receptor complexes prior to molecular dynamics (MD) simulation. AutoDock Vina was used to predict the optimal binding orientation of each ligand at the main binding site of TLR2/1 (36). The ligands were treated as flexible molecules with rotatable bonds. Gasteiger and Kollman charges were assigned, polar hydrogens were added, and both ligand and receptor structures were converted into PDBQT format. The docking grid was defined at the main binding site of TLR2/1, with grid points along the x, y, and z axes and a spacing of 1 Å. To ensure the reliability of our docking parameters, we used crystal-structure-derived agonist coordinates for validation by superimposing them with docking results (32, 33). The low RMSD values obtained confirmed the accuracy of our docking protocol. Additionally, PAM3, as well-established TLR2 agonist, was used as a benchmarking ligand in previous docking studies with AutoDock 4.2 and AutoDock Vina (33, 36, 37). We adopted parameters and genetic algorithm settings from prior literature for AutoDock 4.2 (38, 39) and observed near-identical outcomes from both programs, further affirming the robustness of our approach. Following docking, each ligand–receptor complex was subjected to MD simulation for structural refinement. The MD simulations involved four steps: energy minimization, NVT (constant volume and temperature), NPT (constant pressure and temperature), and production runs in a solvated environment containing Na^+^ and Cl^-^ ions at 140 mM to mimic physiological conditions (39). The system was first minimized using the steepest descent algorithm for 50,000 steps without positional restraints. This was followed by 100 ps of NVT and 100 ps of NPT equilibration with harmonic restraints (1,000 kJ·mol^-1^·nm^-2^) during the NPT phase. Subsequently, a 150-ns production run was performed without restraints to allow the full optimization of receptor–ligand interactions. The TIP3P water model was used, with a minimum solute-box wall distance of 1.5 nm. Simulations were conducted at 300 K using a 2-fs timestep and the LINCS algorithm for bond constraints (40). Periodic boundary conditions were applied throughout all stages. GROMACS 2020 was used for the simulations with CHARMM27 force field parameters (41, 42). Ligand topology files were generated using SwissParam, compatible with CHARMM (43). To estimate binding free energies, the MM/PBSA method was applied using trajectories from the production phase via the gmx MMPBSA tool (44). Snapshots were extracted every 100 ps from the final 10 ns of simulation (140–150 ns), totaling 100 frames for free energy calculations. System stability was confirmed through RMSD analysis. The validity of the MD simulations and MM/PBSA calculations was supported by consistency with our previous studies (32, 33).

### Statistics

The animal was designated as the statistical unit, and data from at least three to five individual replicated experiments (*n* = number of independent cell culture preparations) were presented as mean ± SEM. Error bars were omitted when they fell within the dimensions of the symbols. Statistical analyses were done using SAS software (SAS Institute Inc, USA). One-way analysis of variance (ANOVA) followed by Bonferroni’s post-comparison test with the significance of the data was determined based on their respective *p*-values where *P < 0.05, **P < 0.01, ***P < 0.001, #*P* < 0.05, ##*P* < 0.01, and ### *P* < 0.001.

## Results

### Chemical composition and comparison of the PGN prepared from *Lactobacillus* strains and PGN-Sa

PGN-Lr and PGN-La share the GlcNAc-MurNAc sugar backbone with PGN-Sa as observed in the TLC analysis; however, distinctive structural modifications are found in PGN-Lr by LC/MS analysis. Only intact MurNAc is found in PGN-Sa, whereas O-acetylation is likely in MurNAc residues in PGN-Lr. Both PGN-L and PGN-Sa contain a Lys residue at the stem of the cross-bridge structure, facilitating the linkage between individual PGN chains. Notably, while the cross-bridge in PGN-Sa is composed of Gly residues, a prominent peak in PGN-L in the vicinity of Asp residue at a retention time of 12 min implies that the cross-bridge in PGN-L is a single Asp residue. One of the major differences between PGN-Sa and PGN-L is the bridging residue, which was expected. However, other unexpected residues, such as threonine (Thr), serine (Ser), valine (Val), leucine (Leu), and isoleucine (Ile), were also detected, indicating that possible modifications occurred in the peptide moieties in PGN-L (**Supplementary Figure S2**). However, further comprehensive chemical composition analysis is needed.

### PGN-L decreases PAM3-induced but not LPS-induced pro-inflammatory responses in BEECs

A BEEC viability assay was conducted to investigate the viability of BEECs upon treatment with PGN/PAM3. The results show that the percentage of viable BEECs did not significantly differ among the treatment groups (**Supplementary Figure S3**). However, BEEC viability (**Supplementary Figure S3B**) indicates statistically significant differences between treatment groups at 27 h of incubation time, not a reduction in cytotoxic levels. While treatments such as PAM3 and PGN-Sa led to a modest but statistically significant decrease in cell viability compared to controls, PGN-Lr/La, and pretreated PGN-Lr/La, all groups maintained a viability percentage above 89%–93% (45), which is generally considered acceptable for *in vitro* experiments and unlikely to cause non-specific cytotoxic effects. Therefore, we interpret these changes as biologically minor and insufficient to impair the cells’ overall functionality. Importantly, the observed differences in inflammatory mediator expression are thus more likely due to specific immunomodulatory actions of the compounds rather than a consequence of reduced cell viability. In terms of BEECs’ viability, following LPS treatment, these conditions were carefully selected based on prior studies (20) to ensure physiological relevance while minimizing cellular stress or death.

**Figure 2:**
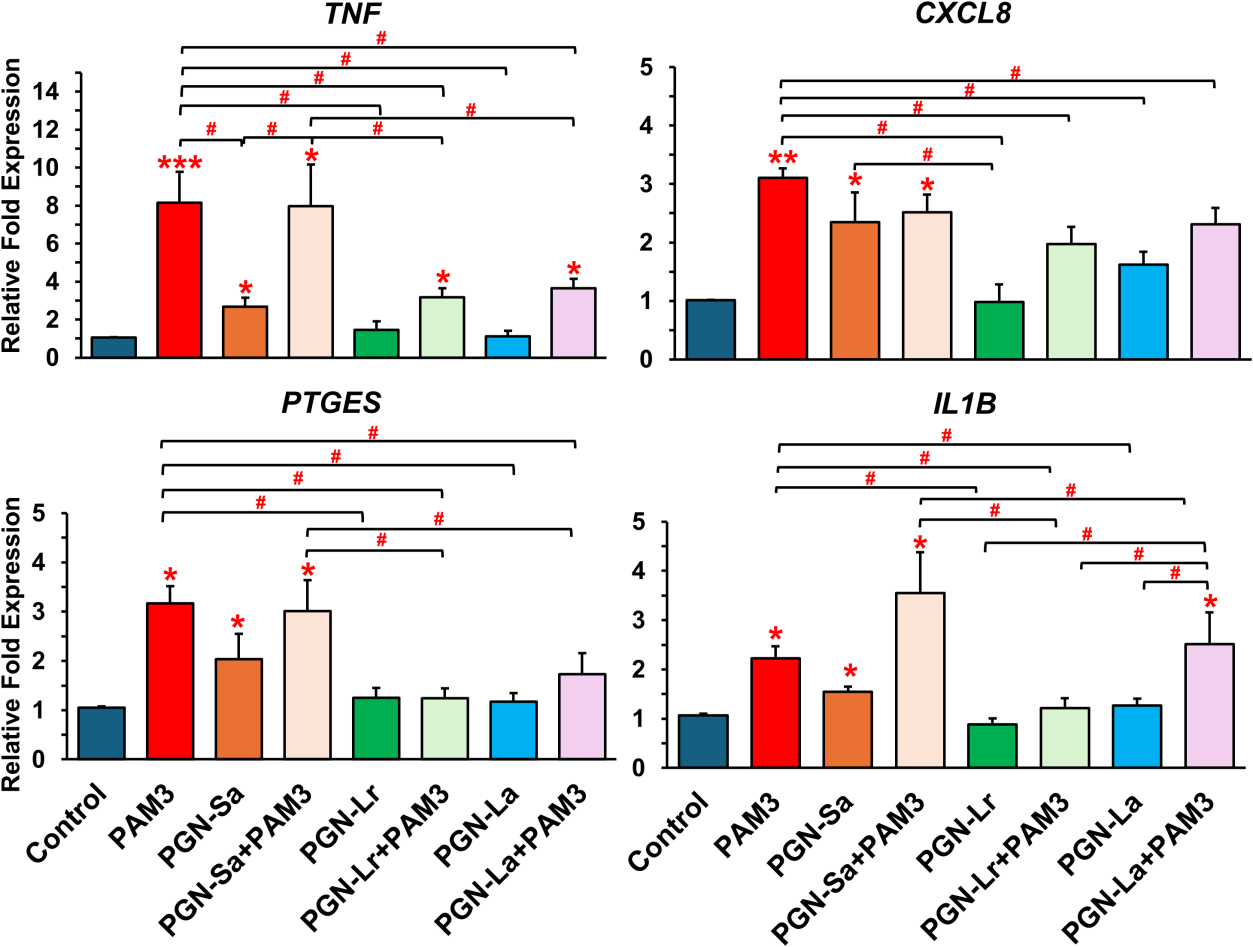
PGN-L (not PGN-Sa) suppresses PAM3-induced inflammatory gene expression in BEECs. BEECs were pretreated with PGN-(Sa, Lr, La) at 1 ng/mL for 24 h. After that, these were combined with stimulated PAM3 at 100 ng/mL for 3 h. The mRNA expression of pro-inflammatory markers (*TNF*, *CXCL8*, *PTGES*, and *IL1B*) was investigated. Data are presented as the mean relative expression ± SEM from five independent experiments. Data were analyzed using one-way ANOVA with Bonferroni’s mean comparisons procedure. The asterisks show a significant difference compared with the control alone. A hash mark is used to show a significant difference compared within the group (*P < 0.05, **P < 0.01, ***P < 0.001, #*P* < 0.05).

**Figure 3:**
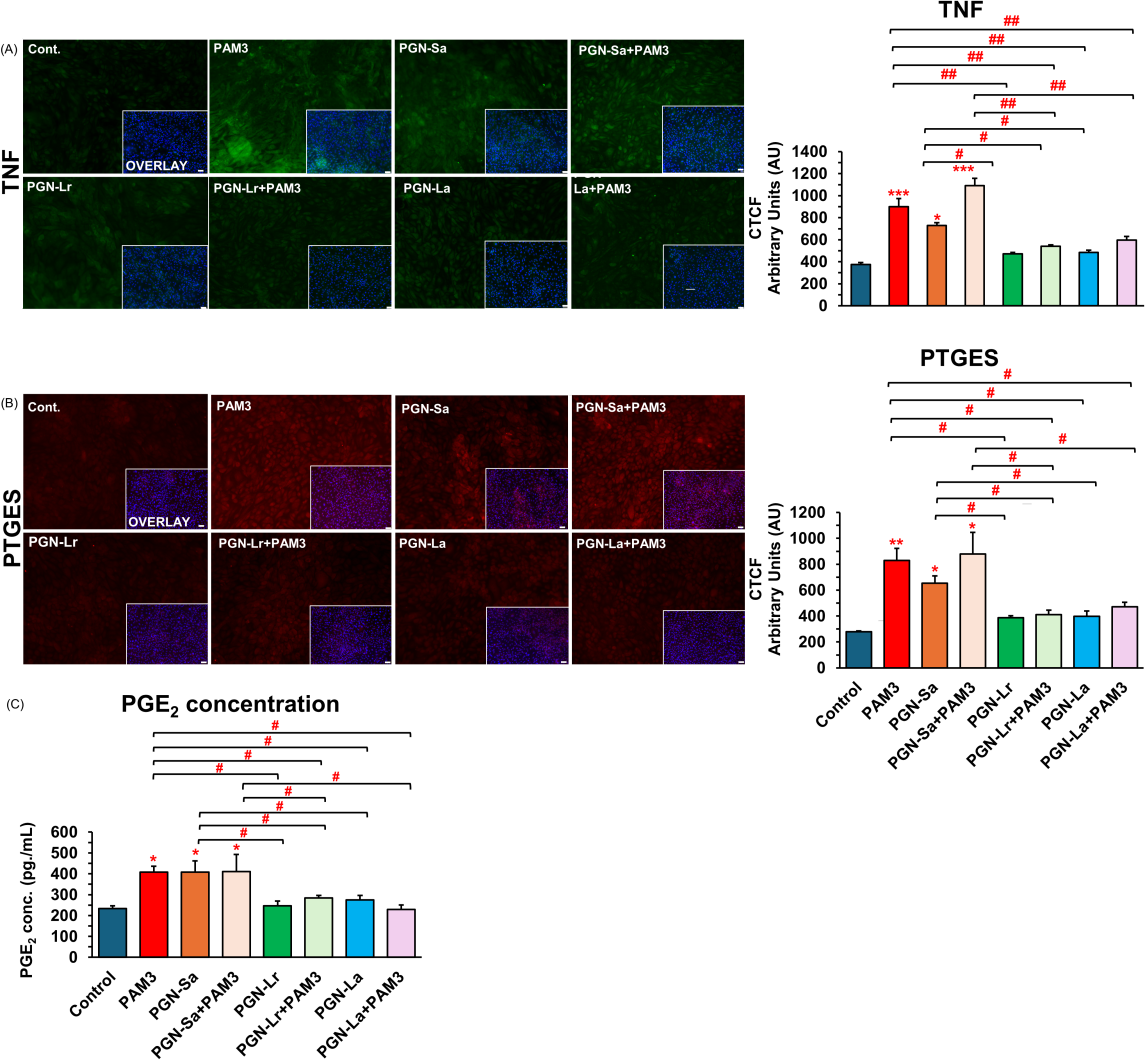
PGN-L (not PGN-Sa) suppresses PAM3-induced inflammatory protein expression in BEECs. BEECs were pretreated with PGN-(Sa, Lr, La) at 1 ng/mL for 24 h. After that, these were combined with stimulated PAM3 at 100 ng/mL for 3 h. BEECs were stained by immunofluorescence staining for TNF and PTGES protein, and BEEC-conditioned media was evaluated for PGE_2_ concentration. **(A)** TNF protein expression (green). **(B)** PTGES protein expression (red). DAPI was set as the nuclear counterstain (blue) in conjunction with a merged image showing DAPI in blue. Semiquantitative scoring of corrected total cell fluorescence (CTCF) of TNF and PTGES protein expressed as arbitrary fluorescence units. **(C)** Secretion of PGE_2_ concentration in BEEC-conditioned media. Data are presented as the mean relative expression ± SEM from four independent experiments. Data were analyzed using ANOVA with Bonferroni’s mean comparisons procedure. The asterisks show a significant difference compared with the control alone. A hash mark is used to show a significant difference compared within the group (*P < 0.05, **P < 0.01, ***P < 0.001, #*P* < 0.05, ##*P* < 0.01, ###*P* < 0.001). Scale bar = 50 μm.

PAM3, PGN-Sa (1 ng/mL), and their combination significantly upregulated the expression of pro-inflammatory genes *TNF*, *CXCL8*, *IL1B*, and *PTGES* in BEECs, while both PGN-Lr (1 ng/mL) and PGN-La (1 ng/mL) did not modulate the pro-inflammatory genes’ expression (**Figure 2**). Pretreatment with PGN-Lr markedly suppressed the PAM3-increased pro-inflammatory gene expression instead. PGN-La pretreatment likewise significantly reduced the expression of *TNF*, *CXCL8*, and *PTGES* pro-inflammatory gene, though it had no significant effect on *IL1B* (**Figure 2**). Furthermore, PAM3 and PGN-Sa elevated the TNF (**Figure 3A**) and PTGES (**Figure 3B**) protein levels, consistent with the mRNA results, whereas PGN-Lr and PGN-La alone did not. Noticeably, both PGN-Lr and PGN-La significantly reduced the PAM3-increased pro-inflammatory protein (TNF and PTGES) expression (**Figures 3A, B**). Moreover, PGN-Lr and PGN-La significantly reduced the PGE_2_ concentration in the conditioned media of PAM3-induced inflammation in BEECs (**Figure 3C**). The low concentrations of PGN-Lr and PGN-La (0.01 and 0.1 ng/mL) did not decrease the PAM3-induced pro-inflammatory gene expression in BEECs (**Supplementary Figure S4**). Besides that, 0.1 ng/mL of PGN-Lr and PGN-La significantly decreased the PAM3-increased *IL1B* mRNA expression (**Supplementary Figure S4B**). LPS at 1 ng/mL for 1 h significantly induced the pro-inflammatory (*TNF*, *CXCL8*, and *IL1B*) and *TLR4* mRNA expression in BEECs. PGN-Lr (high and low doses) did not reduce the LPS-induced pro-inflammatory response (**Supplementary Figure S5**). Moreover, PGN-Lr at 5- and 100-μg/mL pretreatment for 24 h tended to increase the pro-inflammatory (*TNF*, *CXCL8*, and *IL1B*) mRNA expression (but not at low doses).

### PGN modulates TLR2 signaling in BEECs

**Figure 4:**
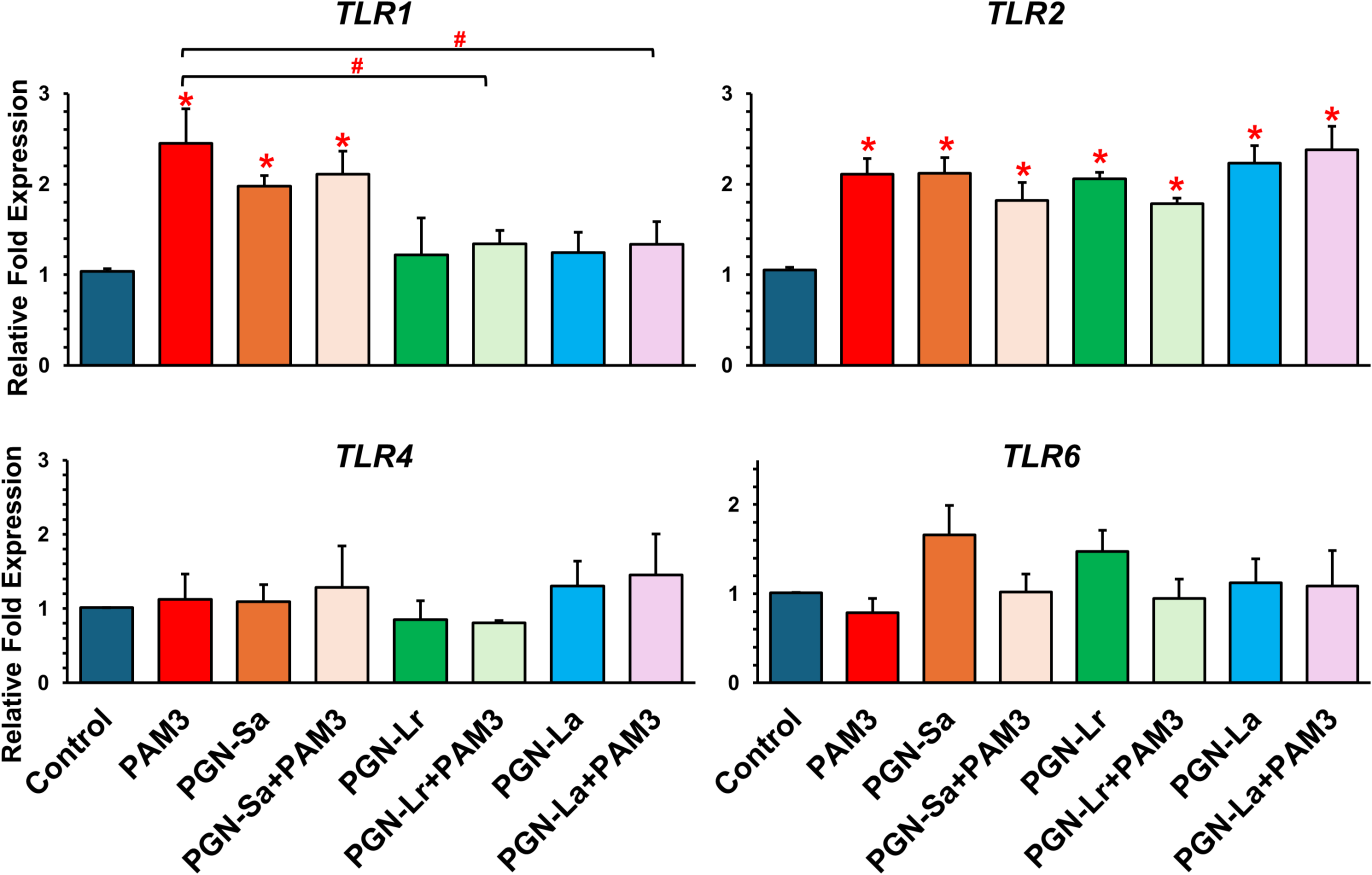
mRNA expression of *TLRs* in BEECs followed by treatment with TLR2 ligands in BEECs. BEECs were pretreated with PGN-(Sa, Lr, La) at 1 ng/mL for 24 h. After that, these were combined with stimulated PAM3 at 100 ng/mL for 3 h. The mRNA expression of TLR2/1-mediated inflammation (*TLR1*, *TLR2*, *TLR4*, and *TLR6*) was investigated. Data are presented as the mean relative expression ± SEM from five independent experiments. Data were analyzed using one-way ANOVA with Bonferroni’s mean comparisons procedure. The asterisks show a significant difference compared with the control alone (*P < 0.05). Hash mark is used to show a significant difference compared within group (#P<0.05).

**Figure 5:**
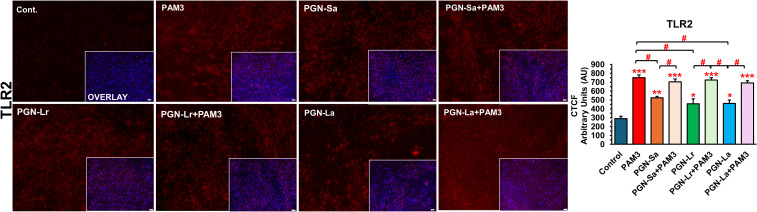
PAM3 and PGN regulate the protein expression levels of TLR2 in BEECs. BEECs were pretreated with PGN-(Sa, Lr, La) at 1 ng/mL for 24 h. After that, these were combined with stimulated PAM3 at 100 ng/mL for 3 h. The protein expression of TLR2 was investigated (red). DAPI was set as the nuclear counterstain (blue) in conjunction with a merged image showing DAPI in blue. Semiquantitative scoring of corrected total cell fluorescence (CTCF) of TLR2 protein expressed as arbitrary fluorescence units. CTCF values are presented as the mean relative expression ± SEM from three independent experiments. Data were analyzed using ANOVA with Bonferroni’s mean comparisons procedure. The asterisks show a significant difference compared with the control alone. A hash mark is used to show a significant difference compared within the group (*P < 0.05, **P < 0.01, ***P < 0.001, #*P* < 0.05). Scale bar = 50 μm.

The mRNA expression levels of *TLR1*, *TLR2*, *TLR4*, and *TLR6* were evaluated in BEECs under various treatment conditions. All treatment groups, including PGN-Sa, PGN-Lr, PGN-La, PAM3, and PGN-PAM3 combinations, significantly upregulated the *TLR2* mRNA expression (**Figure 4**). However, no significant changes were observed in *TLR4* or *TLR6* mRNA expression across the treatments. Notably, PAM3, PGN-Sa, and PAM3 combined with PGN-Sa significantly increased the TLR1 mRNA expression, while PGN-Lr and PGN-La did not alter the TLR1 levels. Moreover, using pretreated PGN-Lr and PGN-La combined with PAM3, the TLR1 mRNA expression was significantly lower than using PAM3 alone (**Figure 4**). At the protein level, using PGN-Sa, PGN-Lr, PGN-La, and PGN, the PAM3 combination treatments significantly enhanced the TLR2 protein expression in BEECs (**Figure 5**). LPS significantly increased the TLR4 expression (**Supplementary Figure S5**).

### PGN-Sa utilizes TLR2/1-mediated inflammation in BEECs, similar to PAM3

**Figure 6:**
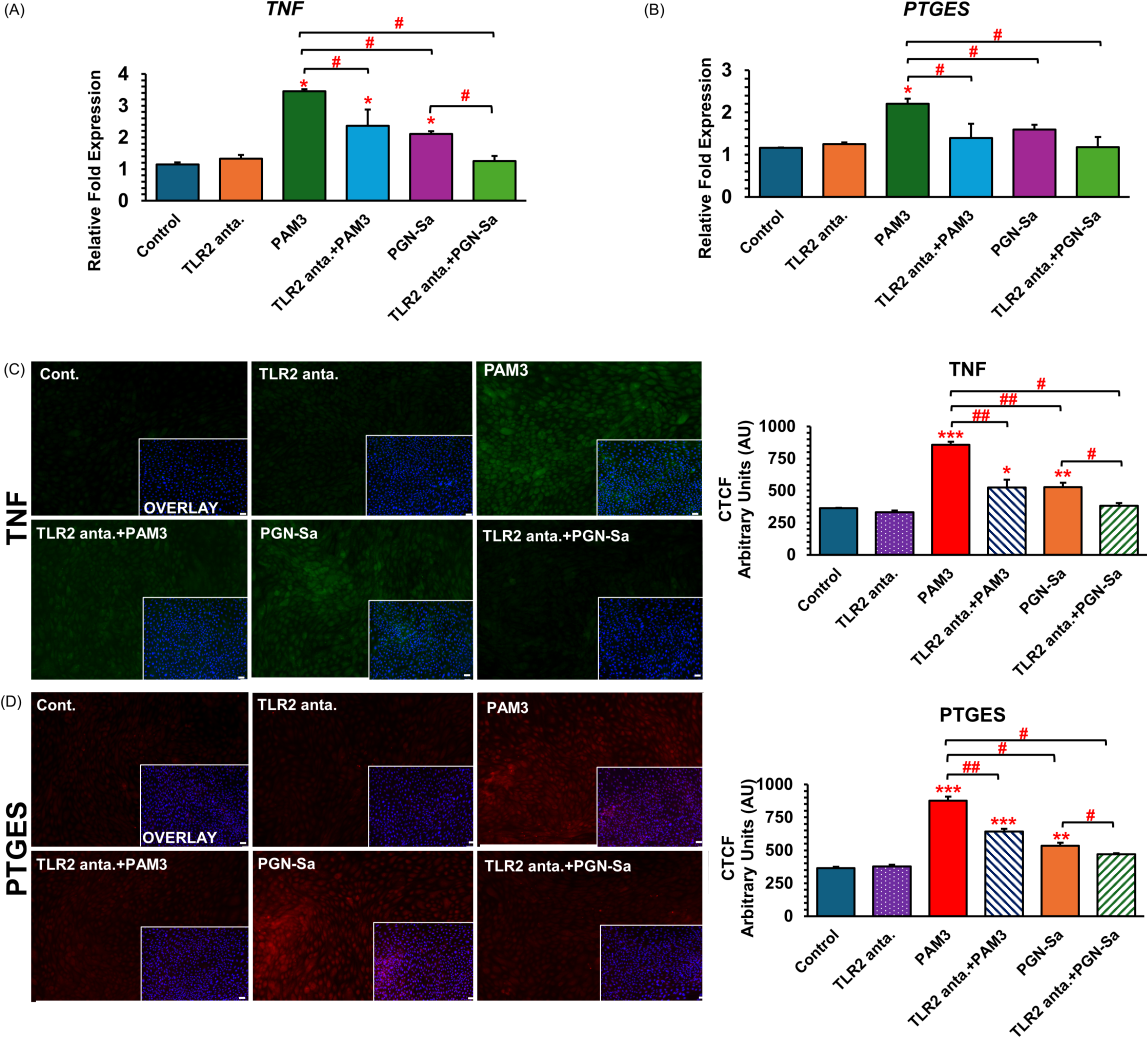
TLR2/1 antagonist blocks PAM3 and PGN-Sa-induced inflammatory markers in BEECs. BEECs were pretreated with TLR2/1 antagonist at 0.1 µM for 3 h. After that, they were combined with stimulated PAM3 at 100 ng/mL for 3 h or PGN-Sa at 1 ng/mL for 24 h. The mRNA expression of pro-inflammatory **(A)** *TNF* and **(B)** *PTGES* and the protein expression of pro-inflammatory **(C)** TNF and **(D)** PTGES were investigated. Data are presented as the mean relative expression ± SEM from three independent experiments. Data were analyzed using one-way ANOVA with Bonferroni’s mean comparisons procedure. The asterisks show a significant difference compared with the control alone. A hash mark is used to show a significant difference compared within the group (*P < 0.05, **P < 0.01, ***P < 0.001, #*P* < 0.05, ##*P* < 0.01).

Pretreatment with a TLR2/1 antagonist and stimulation with either PGN-Sa or PAM3 revealed a modulatory effect on inflammatory responses *TNF* and *PTGES* mRNA (**Figures 6A, B**) and protein expression levels (**Figures 6C, D**) in BEECs. Specifically, stimulation with PGN-Sa or PAM3 alone significantly increased the pro-inflammatory markers’ expression levels, but TLR2/1 antagonist alone did not modulate. BEECs pretreated with the TLR2/1 antagonist before PGN-Sa or PAM3 exposure altogether exhibited markedly reduced inflammatory markers (TNF and PTGES), suggesting that PGN-Sa or PAM3 utilized the TLR2/1 signaling pathway.

### PGN-S and PAM3 bind to the TLR2/1 dimer, while PGN-L exhibits no interaction

**Figure 7:**
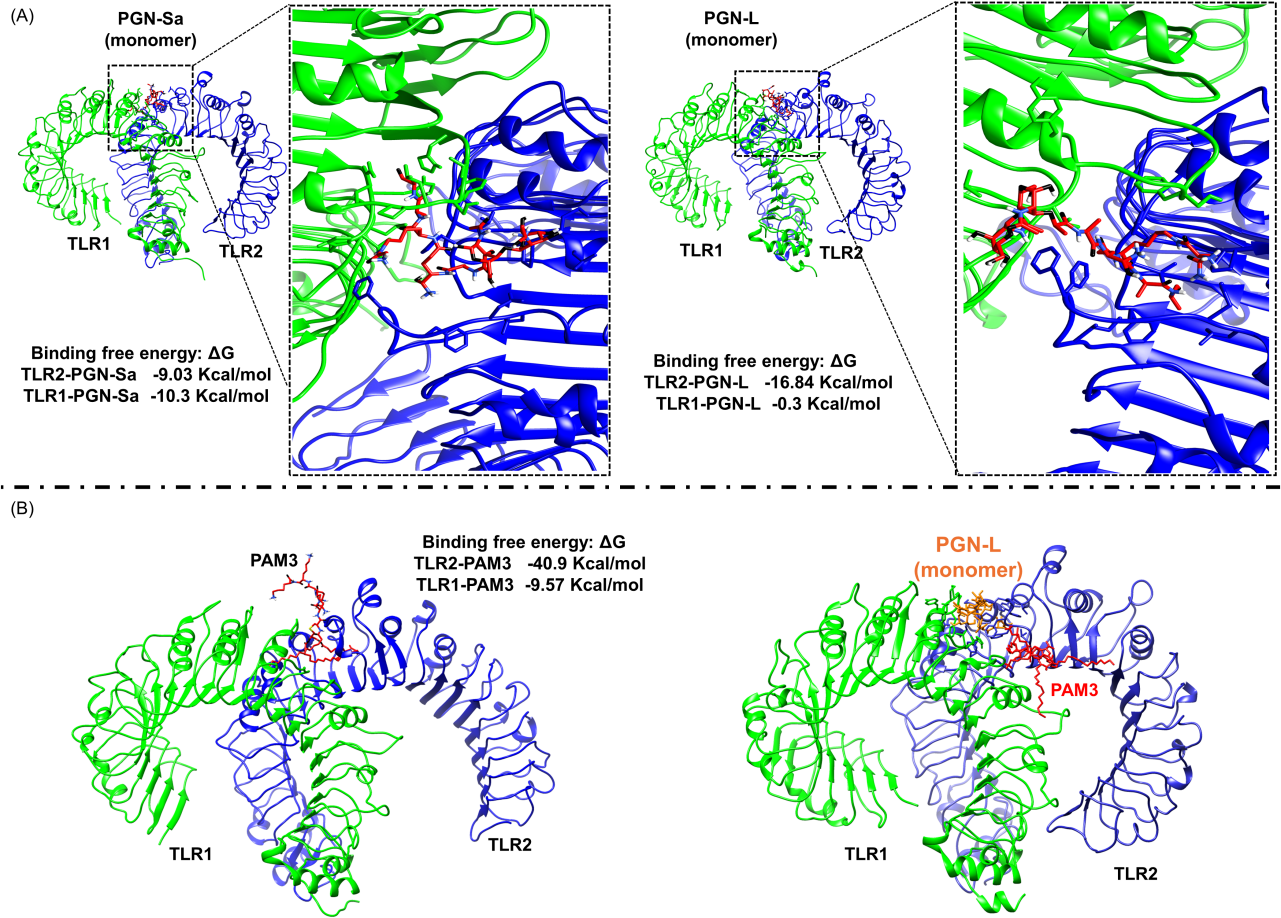
Computational analysis reveals that PGN-L, unlike PGN-S, interferes with PAM3 binding to the TLR2/1 complex. **(A)** *In silico* data indicate that PAM3 and PGN-S can stabilize TLR2/1 dimerization, whereas PGN-L does not, based on binding free energy (BFE) calculations for PGN-Sa–TLR2/1 and PGN-L–TLR2/1 complexes. TLR2 and TLR1 are colored blue and green, respectively. **(B)** PGN-L occupies the main binding pocket of TLR2 (but not TLR1), thereby inhibiting PAM3-mediated TLR2/1 dimerization.

Computational analysis revealed that PGN-Sa interacts with TLR2/1 dimerization, similar to PAM3. On the contrary, PGN-L binds TLR2 (but not TLR1) only. Calculations using the gmx_MMPBSA tool showed that the BFE between PGN-Sa demonstrated comparable interactions: the BFE between PGN-Sa and TLR2 was -9.03 kcal/mol, and with TLR1 it was -10.3 kcal/mol, as shown in **Figure 7A** (left). Following PAM3, the BFE between PAM3 and TLR2 was -40.9 kcal/mol, and between PAM3 and TLR1 it was -9.57 kcal/mol, indicating a highly favorable interaction with the dimer, as shown in **Figure 7B** (left). As expected, it interacts strongly with both TLR2 and TLR1 within the TLR2/1 dimer, supporting its role in promoting dimerization. These values suggest that PGN-Sa can also engage both receptors and may support TLR2/1 dimerization. In contrast, PGN-L showed a distinct interaction profile. It could bind moderately to TLR2 with a BFE of -16.84 kcal/mol but exhibited almost no interaction with TLR1 (BFE: -0.3 kcal/mol), as shown in **Figure 7A** (right). These findings suggest that while PAM3 and PGN-Sa may function as ligands or agonists promoting TLR2/1 dimerization, PGN-L likely acts as a blocker, preventing this dimer formation by failing to engage TLR1. To investigate whether PGN-L acts as a blocker or antagonist of the TLR2/1 dimer, we performed a computational analysis by docking PAM3 to the TLR2/1-PGN-L complex, followed by MD simulation. The results indicated that PGN-L can obstruct PAM3 from accessing the TLR2/1 binding site. As shown in **Figure 7B** (right), PGN-L occupies the main binding pocket of TLR2, thereby preventing effective PAM3 binding and ultimately inhibiting TLR2/1 dimerization.

## Discussion

This study integrated *in vitro* cellular assays with *in silico* molecular modeling to elucidate the differential immunomodulatory effects of peptidoglycans (PGNs) derived from distinct Gram-positive bacterial species on the bovine endometrium. Our key findings reveal that pretreatment with PGNs isolated from lactic acid bacteria (*Lacticaseibacillus rhamnosus* and *Lactobacillus acidophilus*) (PGN-L) significantly reduced the activation of the TLR2/1 heterodimer (induced by PAM3CSK4:PAM3), but not TLR4 (induced by LPS), in BEECs subjected to inflammatory stimuli. Notably, we demonstrated that PGN-L pretreatment effectively suppressed the expression of PAM3-induced pro-inflammatory mediators in BEECs. Pretreated PGN-Lr and PGN-La combined with PAM3 significantly increased TLR2 (but not TLR1), suggesting a potential mechanism for dampening uterine inflammatory responses by disturbing TLR2/1 dimerization (increased *TLR2* mRNA expression but not TLR1).

The bovine uterus exhibits a dynamic immune environment where TLR2/1 signaling plays a dual role. We have previously shown that sperm triggers a physiological inflammatory response in the bovine uterus via TLR2/1 (18, 27), a process distinct from the pathological inflammation induced by bacterial infection and their products, which can lead to endometritis and substantial economic losses (46). Notably, TLR2 is not solely involved in inflammation but also plays a crucial role in maintaining the homeostasis of pro- and anti-inflammatory immune responses in epithelial cells and in protecting against epithelial injury (17). Bacterial cell wall components like LPS, PGN, and lipoproteins are key ligands for TLRs, with LPS being recognized by TLR4 and PGN and lipoproteins primarily by TLR2 (10). The synthetic lipopeptide PAM3 is a well-established mimic of lipoprotein-activated TLR2/1 dimerization, potently inducing inflammation (27).

While LPS from Gram-negative bacteria is a significant contributor to bovine endometritis (46), PGN-Sa, a component of the pathogenic Gram-positive bacterium *S. aureus*, exerts multifaceted effects on the reproductive system. Beyond its pro-inflammatory activity in BEECs via TLR2 (11), PGN-Sa has been reported to inhibit the production of crucial reproductive hormones, progesterone (P4) and androstenedione (A4), in bovine theca cells (21, 47), potentially disrupting endocrine balance. Interestingly, PGN-Sa has also been shown to suppress the sperm-induced physiological inflammation in BEECs (11), highlighting its complex immunomodulatory capacity. Conversely, PGN derived from lactic acid bacteria (PGN-L) has demonstrated different immunomodulatory profiles in other systems, including the inhibition of LPS-induced inflammation (16) and the upregulation of avian defensin B-9 in avians (15).

In the context of the bovine endometrium, our findings indicate that PGN-L does not directly elicit a strong pro-inflammatory response. However, it demonstrates a significant capacity to interact with the TLR2 pathway, specifically by attenuating PAM3-induced inflammation without affecting TLR4 signaling. Although PGN-Lr alone did not suppress LPS-induced pro-inflammatory mRNA expression of *TNF* and *CXCL8* in BEECs, it is important to consider that whole LAB, including *L. rhamnosus*, are known to produce a range of anti-inflammatory substances and can modulate host immune responses through various mechanisms (48). Furthermore, the potential of LAB combinations, such as *L. rhamnosus*, as probiotics to mitigate *E. coli*-associated uterine inflammation (endometritis) in dairy cows is gaining recognition (49), suggesting a promising alternative to conventional antibiotics.

The observed ability of PGN-L to specifically attenuate PAM3-induced (TLR2/1-mediated) pathological inflammation in BEECs, while not affecting LPS-induced (TLR4-mediated) inflammation in our specific experimental setup, is a key finding. It is noteworthy that in other cellular contexts, higher concentrations of PGN-L have been reported to suppress LPS-induced inflammation (16), suggesting that the immunomodulatory effects of PGN-L may be cell type and concentration dependent. In our initial experiments, we tested PGN-Lr (*L. rhamnosus*) with LPS (TLR4 ligand) and observed no significant effect, suggesting the limited involvement of TLR4. However, PGN-Lr showed a clear response with PAM3 (TLR2/1 ligand), indicating a TLR2/1-dependent effect. Based on this finding, we focused on the TLR2 pathway and tested PGN-La (*L. acidophilus*) only in combination with PAM3. According to our chemical analysis, PGN-Lr and PGN-La share highly similar structures, and both showed similar functional effects when combined with PAM3. Therefore, we expect that PGN-La would likely behave similarly to PGN-Lr in the context of TLR4. The reduction in key pro-inflammatory markers (TNF, PTGES, IL1B, and CXCL8) in response to PAM3 stimulation following PGN-L pretreatment supports the concept that PGN-L possesses immunomodulatory properties capable of mitigating excessive inflammatory responses in BEECs, a crucial aspect of endometritis pathogenesis in dairy cows (50). This aligns with existing literature highlighting the anti-inflammatory effects of various *Lactobacillus* species and their cell wall components in different epithelial and immune cell types (6, 51).

Besides that, the extracted PGN-Lr and PGN-La were purified using organic solvents such as methanol and chloroform, which effectively removed non-covalently bound lipophilic materials, including membrane residues and wall-associated polymers. This method ensures the selective isolation of the peptidoglycan sacculus with minimal contamination. In contrast, despite differences in the purification strategies, the commercial PGN-Sa underwent detergent-based purification procedures to remove residual lipoproteins and other hydrophobic substances commonly associated with Gram-positive bacterial envelopes. However, the chemical analysis revealed that the glycan backbone and amino acid components of PGN-Lr or PGN-La and PGN-Sa exhibited comparable profiles (**Supplementary Figure S2**), with overlapping peaks indicating structural similarity in their core muropeptide compositions. This suggests that while purification methods target different impurities, they preserve the fundamental peptidoglycan architecture necessary for comparative structural and immunological analyses. The main structural difference between PGN-L and PGN-S lies in the peptide chain, not the carbohydrate portion. While PGN-Lr showed an additional spot in the glycan chain compared to PGN-La, the similar immune responses observed suggest that this variation is not functionally significant.

To address why different PGNs (PGN-L and PGN-Sa) exhibit differential functions in the bovine endometrium through the TLR2/1 system, a chemical analysis was performed, followed by computational experiments. Our findings indicated that PGN-L and PGN-Sa differ significantly in their structural composition, which may underlie their distinct immunomodulatory effects. PGN-L typically consists of N-acetylglucosamine (GlcNAc) and N-acetylmuramic acid (MurNAc) units linked with peptide chains rich in Lys, commonly found in Gram-positive commensal bacteria such as *Lactobacillus* species (35). In contrast, PGN-Sa, derived from the pathogenic *S. aureus*, contains a similar glycan backbone but features interpeptide bridges composed of pentaglycine, contributing to its structural rigidity and potent immune-stimulating properties (52). These compositional differences may influence pattern recognition receptor (PRR) interactions, particularly with TLRs, resulting in differential activation of inflammatory pathways. Understanding these structural variations is critical for interpreting their biological effects and designing targeted immunotherapies. On the one hand, TLC analysis clarified the presence of GlcNAc and MurNAc in all of the PGN samples. Low mobility spot with tailing was observed in all of the samples, indicating that the modification occurred. However, a yellow high mobility spot was observed only in PGN-Lr. This might be a result of the modification in the PGN-Lr; therefore, further analysis is needed. In general, the major peak pattern of all the PGN samples was very similar. The presence of a significant amount of Lys residues indicated that PGN-Sa, PGN-La, and PGN-Lr were the Lys-type PGN (35, 53). Furthermore, the relatively higher content of Gly in PGN-Sa indicated that the bridge structure was constituted by Gly residues. The evaluated molar ratio of the major amino acid residues in PGN-Sa was Glu/Gly/Ala/Lys (0.169:0.47:0.366:0.17), respectively, suggesting that the number of bridge-forming Gly residues were two to three. On the other hand, there was a major peak eluted out at around 12 min in PGN-Lr and PGN-La. Normally, Asp residue should be eluted out in this area (35), but the peak in our samples was not assigned as such. Therefore, further investigation is needed even though a single Asp residue has a high potential to form a crossbridge as previously reported. Finally, we concluded that the purity of PGN-L was enough for biological assay because several minor peaks observed in PGN-L were also detectable in PGN-Sa.

*In silico* data revealed distinct TLR binding preferences between PGN-Sa and PGN-L, which may explain their divergent immunological outcomes. PGN-Sa demonstrated a strong binding affinity to the TLR2/1 heterodimer, like PAM3, suggesting that it can activate pro-inflammatory signaling through the TLR2/1 pathway. In contrast, PGN-L preferentially interacted with TLR2 alone, without stable engagement with TLR1, indicating as TLR2 antagonist. To activate signaling through TLR2/1, the heterodimer must be stabilized, which is the role of a ligand or agonist. These molecules interact strongly with both TLR2 and TLR1, forming a bridge between them that stabilizes the complex and initiates TLR2/1 signaling. In contrast, antagonist molecules may occupy the binding site of TLR2 and/or TLR1 but do not bridge the two receptors, thereby preventing activation (27). This selective binding could underlie PGN-L’s ability to modulate inflammation without inducing the whole pro-inflammatory cascade typically triggered by TLR2/1 activation. These results are consistent with previous studies emphasizing ligand-specific recognition by TLR complexes and highlight the potential of PGN-L as a targeted immunomodulator with reduced inflammatory risk (54, 55).

PAM3 is an agonist molecule that promotes heterodimerization between TLR2 and TLR1, initiating the downstream signaling pathway (27, 33). To investigate the role of PGN-Sa in this dimerization process, we conducted two types of experiments: *in vitro* and *in silico*. For the *in vitro* study, we utilized a TLR2/1 antagonist. In our previous work, we demonstrated that this antagonist is a crucial molecule capable of binding to the main site shared by both TLR2 and TLR1, thereby blocking access for PAM3 (33). Our *in vitro* results showed that the antagonist was also able to inhibit the interaction between PGN-Sa and the TLR2/1 complex, suggesting that PGN-Sa behaves similarly to PAM3. Additionally, our computational analysis revealed that PGN-Sa, but not PGN-L, can form a bridge between TLR2 and TLR1, promoting their dimerization. These findings suggest that PGN-Sa, like PAM3, enhances TLR2/1 dimerization, whereas PGN-L may inhibit this process. Our primary aim was to establish an initial evidence of the differential immunomodulatory effects of PGN-Lr and PGN-La in a physiologically relevant model using BEECs. To further deepen the study, future experiments using HEK cells overexpressing specific receptors such as TLR2/1 and NODs (56) could help clarify the recognition mechanisms of different PGNs. Such receptor-specific assays would offer valuable mechanistic insights. In addition, *in vivo* studies are essential to supplement this finding.

**Figure 8:**
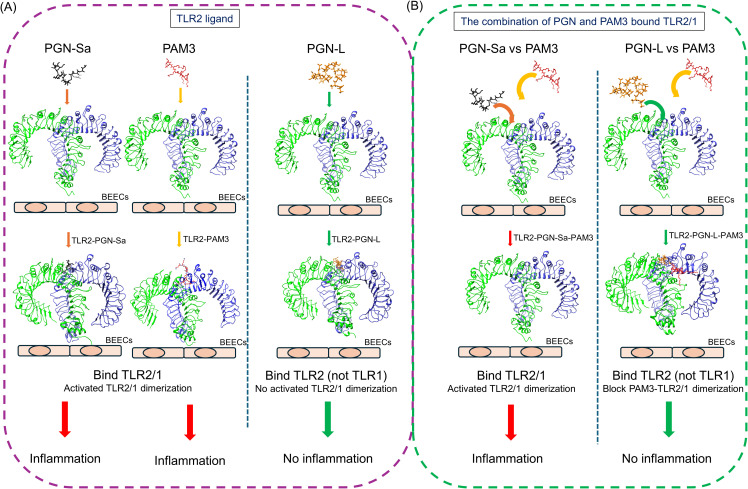
Schematic illustration showing the disruption of PAM3-activated TLR2/1 dimerization by PGN-L. **(A)** The mimicking of PGN-Sa, PGN-L, PAM3, and TLR2/1 heterodimers show that PGN-Sa and PAM3 stabilize TLR2/1 dimerization and lead to inflammation. On the contrary, PGN-L cannot regulate TLR2/1 dimerization which is not able to induce inflammation. **(B)** Furthermore, PGN-L disturbs PAM3-activated TLR2/1 dimerization (but not PGN-Sa). In conclusion, PGN-L potentially modulates the TLR2 receptor as a blocker to inhibit PAM3-induced inflammation in BEECs.

In conclusion, our findings suggest a novel immunomodulatory mechanism whereby peptidoglycans derived from *Lactobacillus* species selectively interact with TLR2, hindering its ability to heterodimerize with TLR1. This disruption of TLR2/1 complex formation effectively antagonizes PAM3-induced pro-inflammatory signaling in BEECs (**Figure 8**). This highlights the intricate and receptor-specific interactions between distinct microbial components and host immune receptors, revealing potential therapeutic strategies for modulating inflammatory responses. While our *in vitro* data provide compelling evidence for this mechanism, further investigation is warranted to fully elucidate the precise molecular interactions between PGN-L and TLR2, including the specific binding sites and downstream signaling pathways affected. The observed capacity of PGN-L to selectively reduce TLR2/1-mediated inflammation suggests its potential to contribute to uterine homeostasis and mitigate fertility issues associated with chronic inflammation. Consequently, future *in vivo* studies are crucial to validate these findings within the complex bovine uterine environment and to explore the therapeutic efficacy of PGN-L as a preventative or supplementary treatment for uterine inflammatory conditions.

## Data Availability

The original contributions presented in the study are included in the article/**Supplementary Material**. Further inquiries can be directed to the corresponding author.
