# Supplementary material for: Peptidoglycan derived from Lacticaseibacillus rhamnosus and Lactobacillus acidophilus suppress TLR2/1-mediated inflammation in bovine endometrial epithelial cells
Source: Front Immunol. 2025 Jun 18;16:1622307. doi: 10.3389/fimmu.2025.1622307 (PMC12213376; doi:10.3389/fimmu.2025.1622307)
Supplement: Supplementary file 1 [file DataSheet1.docx]

**Supplementary Material**

**Peptidoglycan Derived from *Lacticaseibacillus rhamnosus* and *Lactobacillus acidophilus* Suppresses TLR2/1-Mediated Inflammation in Bovine Endometrial Epithelial Cells**

**Elham Waehama^1,2^, Kenji Fukuda^1^, Alireza Mansouri^1^, Malinda Hulugalla^1^, Ihshan Akthar^1^, Mohamed Samy Yousef^1,3^ and Akio Miyamoto^1*^**

*^1)^Global Agromedicine Research Center (GAMRC), Obihiro University of Agriculture and Veterinary Medicine, Obihiro, Japan*

*^2)^Faculty of Agriculture, Princess of Naradhiwas University, Narathiwat, Thailand*

*^3)^Department of Theriogenology, Faculty of Veterinary Medicine, Assiut University, Assiut, Egypt*

**Keywords**: Peptidoglycan, *Lactobacillus* species, *Staphylococcus aureus*, PAM3, inflammation, bovine endometrial epithelial cells

**Running head:** *Lactobacillus*-peptidoglycan on bovine uterine inflammation

***Correspondence**

Akio Miyamoto

[akiomiya@obihiro.ac.jp](mailto:akiomiya@obihiro.ac.jp)

TEL +81-155-49-5416/FAX +81-155-49-5593

1. **Supplementary data:**

*In silico*, the structure of PGN-L and PGN-Sa were constructed following to the chemical details illustrated (32,33) (Supplementary Figure 1). Chemical analysis revealed that the key difference between PGN-Sa and PGN-L is the bridging residue. In PGN-Sa, Gly occupies the bridging position, whereas in PGN-L, it is Asp, as expected. The main structure between PGN-L and PGN-Sa lies in their peptide and backbone regions. Specifically, after the Ala residue, PGN-Sa contains Gln, whereas PGN-L contains Glu. In the backbone, PGN-Sa features a Gly residue, while PGN-L includes Asp.

1. **Supplementary data:**

The carbohydrate and amino acid composition of PGN was determined by thin-layer chromatography (TLC) and amino acid analysis, respectively. PGN derived from *S. aureus* was used as the reference substance. PGN-Lr and PGN-La share the GlcNAc-MurNAc sugar backbone with PGN-Sa (Supplementary Figure 2A). Both PGN-L and PGN-Sa contain Lys and Gly residues. In PGN-Lr and PGN-La, presumably modified amino acid residues of the cross-bridge (Supplementary Figure 2B).

1. **Supplementary data:**

BEECs viability assay was conducted to investigate the viability of BEECs upon treatment with PGN/PAM3. The results, illustrated in the bar graph, show that the percentage of viable BEECs did not significantly differ among the treatment groups (Supplementary Figure 3A). BEEC viability decreased over time in all treatment groups, with the most prominent reductions observed in PGN-Sa+PAM3 and PGN-Sa groups. According to the line graph (Supplementary Figure 3B), all groups exhibited a slight decline from baseline 0 h to 24 h, followed by a further reduction at 27 h. The control group maintained relatively stable viability across time, indicating that the experimental conditions alone did not affect cell viability. In contrast, groups treated with peptidoglycan (PGN) and PAM3 combinations showed a significantly steeper decline, particularly PGN-Sa+PAM3 at 27 h compared with control. However, neither PGN-Lr nor PGN-La reduced the percentage of BEEC viability over time.

1. **Supplementary data**

BEECs were pretreated with PGN (Sa, Lr, La) at 0.01 or 0.1 for 24 h, they were then stimulated with PAM3 100 ng/mL for 3 h. The pro-inflammatory mRNA expression (*TNF, CXCL8, IL1B* and *PTGES*), and pattern recognition receptors (PRRs) including TLRs family (TLR1, TLR2, TLR4, and TLR6) was investigated. The results illustrated that PGN-L and PGN-Sa at 0.01 and 0.1 ng/mL for 24 h did not decrease PAM3-induced pro-inflammatory mRNA expression in BEECs (Supplementary Figure 4A, 4B). Furthermore, PGN (Lr, La, Sa) pretreatment with (0.01, 0.1 ng/mL) did not upregulate TLRs mRNA expression (Supplementary Figure 4A, 4B). Besides, 0.1 ng/mL of PGN-Lr and PG-La significantly decreased PAM3-increased *IL1B* mRNA expression (Supplementary Figure 4B).

1. **Supplementary data**

BEECs were pretreated with PGN-Lr (high/low doses) for 24 h, they were then stimulated with PAM3 100 ng/mL for 3 h. The pro-inflammatory mRNA expression (*TNF* and *CXCL8*) was investigated as well as Toll-like receptor 4 (*TLR4*). To evaluate the impact of PGN-Lr on LPS-induced inflammation in BEECs. The results illustrated that PGN-Lr at 5 and 100 μg/mL for 27 h incubation significantly increased *TNF* and *CXCL8* mRNA expression (Supplementary Figure 5A). However, *TLR4* was significantly increased by LPS only. Furthermore, pretreated PGN-Lr (high/low doses) for 24 h did not decrease LPS-induced pro-inflammatory mRNA expression in BEECs (Supplementary Figure 5).


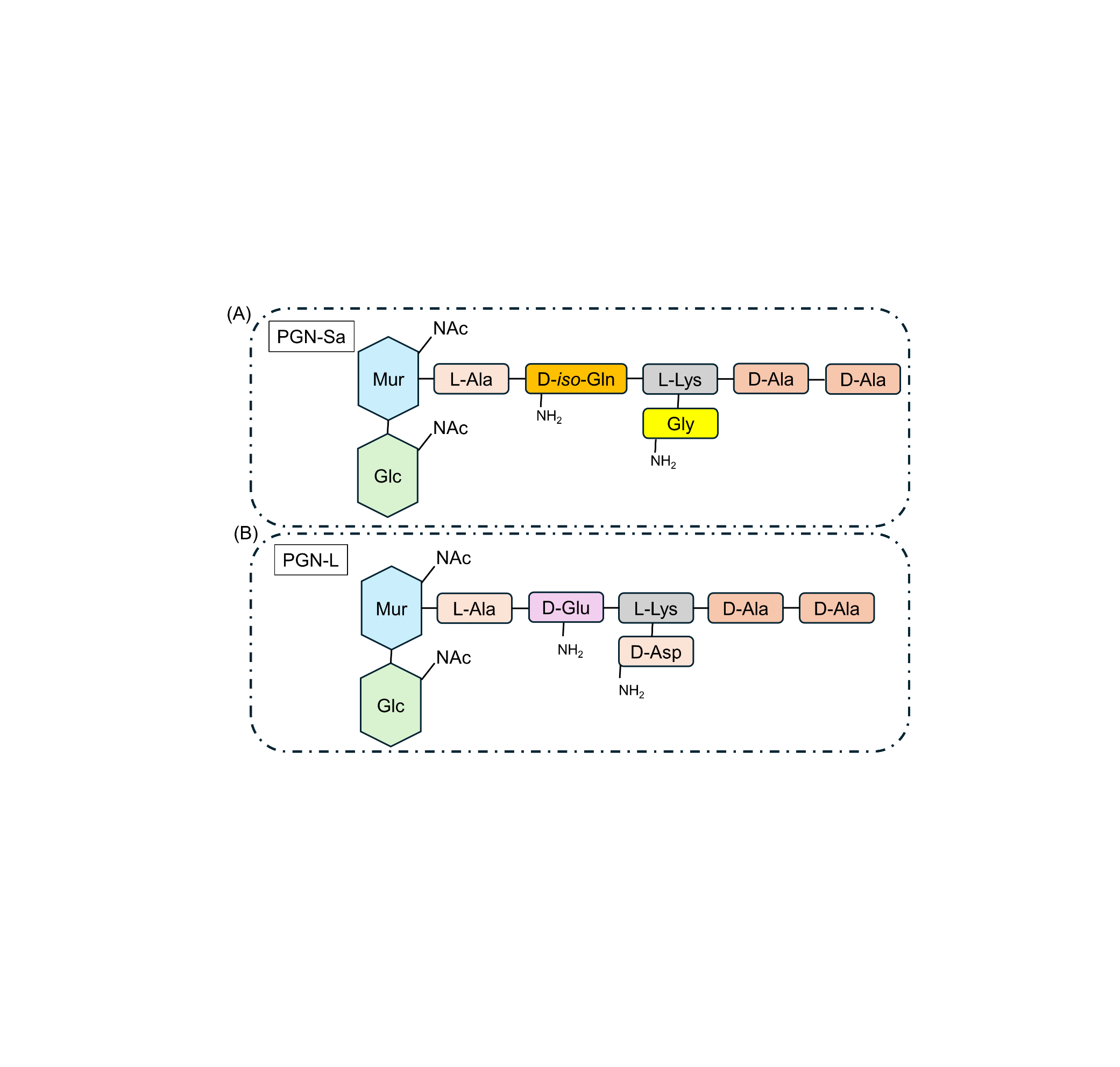


**Supplementary Figure 1. The schematic representation of the general structure of PGN.** **(A)** The illustration of *Staphylococcus* aureus-derived peptidoglycan (PGN-Sa), and **(B)** Lactic acid bacteria-derived peptidoglycan, which are comprised of glycan and amino acid peptide.


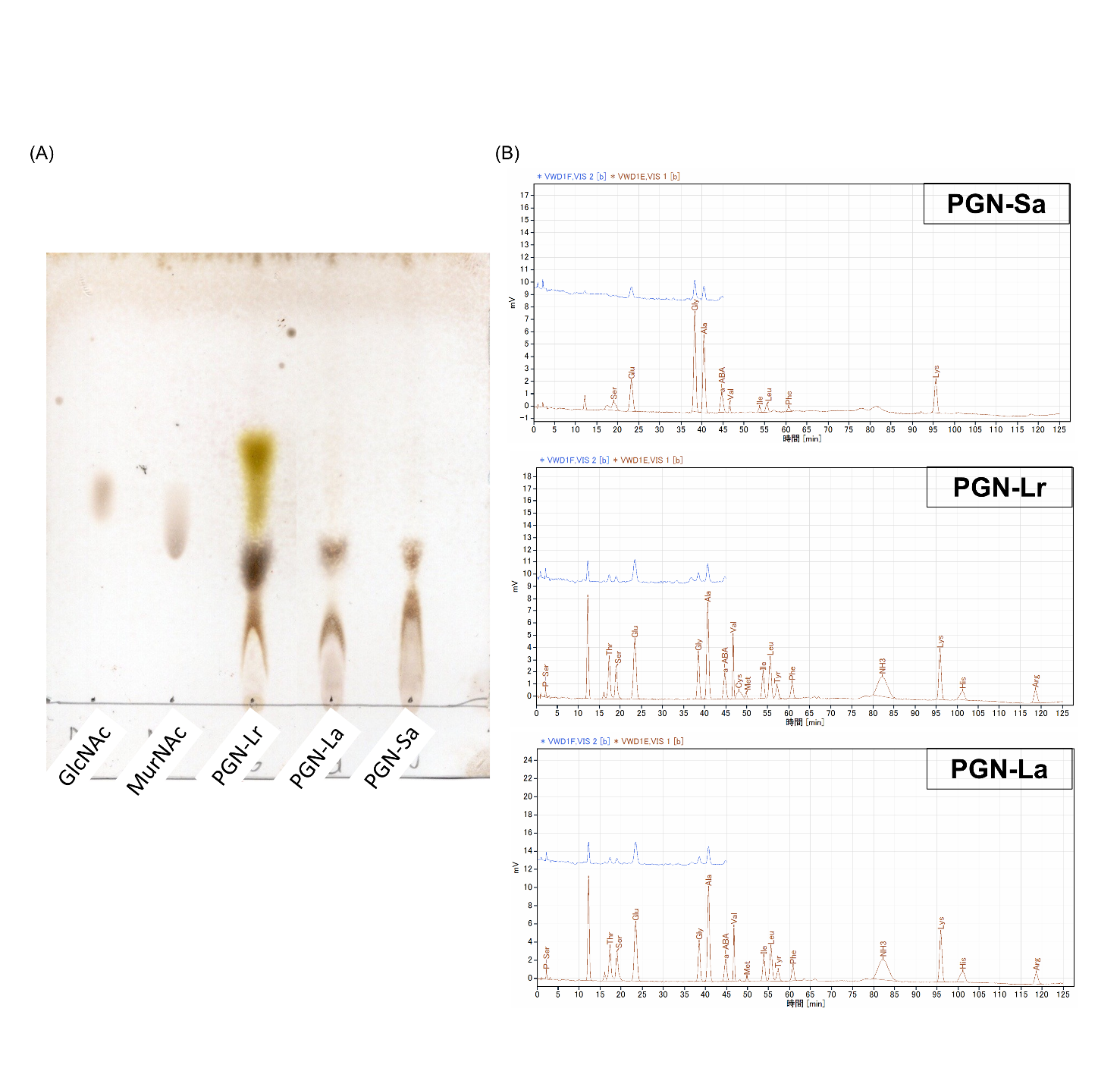
**Supplementary Figure 2.** **The composition of PGN-L and PGN-Sa analysis results. (A)** Carbohydrate composition of PGN analyzed on TLC. **(B)** Amino acid composition of PGN analyzed by amino acid analyzer.


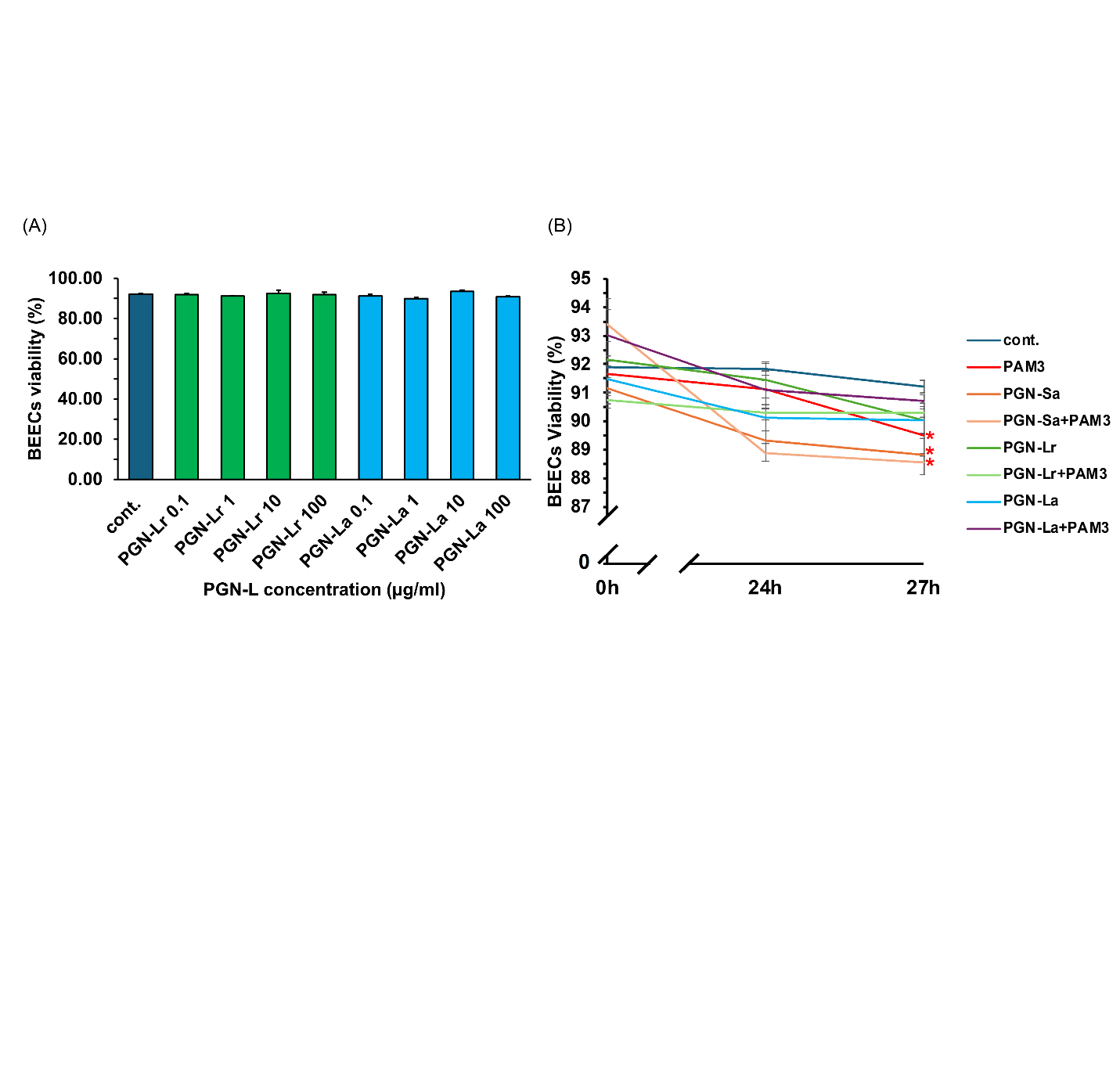


**Supplementary Figure 3.** **The impact of PGN/PAM3 on the BEEC viability** **(A)** BEECs were challenged with PGN-Lr and PGN-La (0.1, 1, 10, 100 μg/mL) 30 h, and **(B)** BEECs were pretreated PGN (Sa, Lr, La) 1 ng/mL for 24 h., then were stimulated PAM3 100 ng/mL for 3 h. At the end of the experiment, BEEC viability was examined at 0, 24, and 27 h of incubation time. Data are presented as the mean of the BEECs viability ± SEM percentage from 4 independent experiments. Statistical analysis was performed using one-way and two-way repeated measures ANOVA to evaluate the effects of treatment group, time, and their interaction on BEECs viability. Post-hoc comparisons were conducted using Bonferroni’s post-comparison test to determine significant differences between groups at each time point. Asterisks show a significant difference compared with the control alone in the single time point. (*P< 0.05).

**
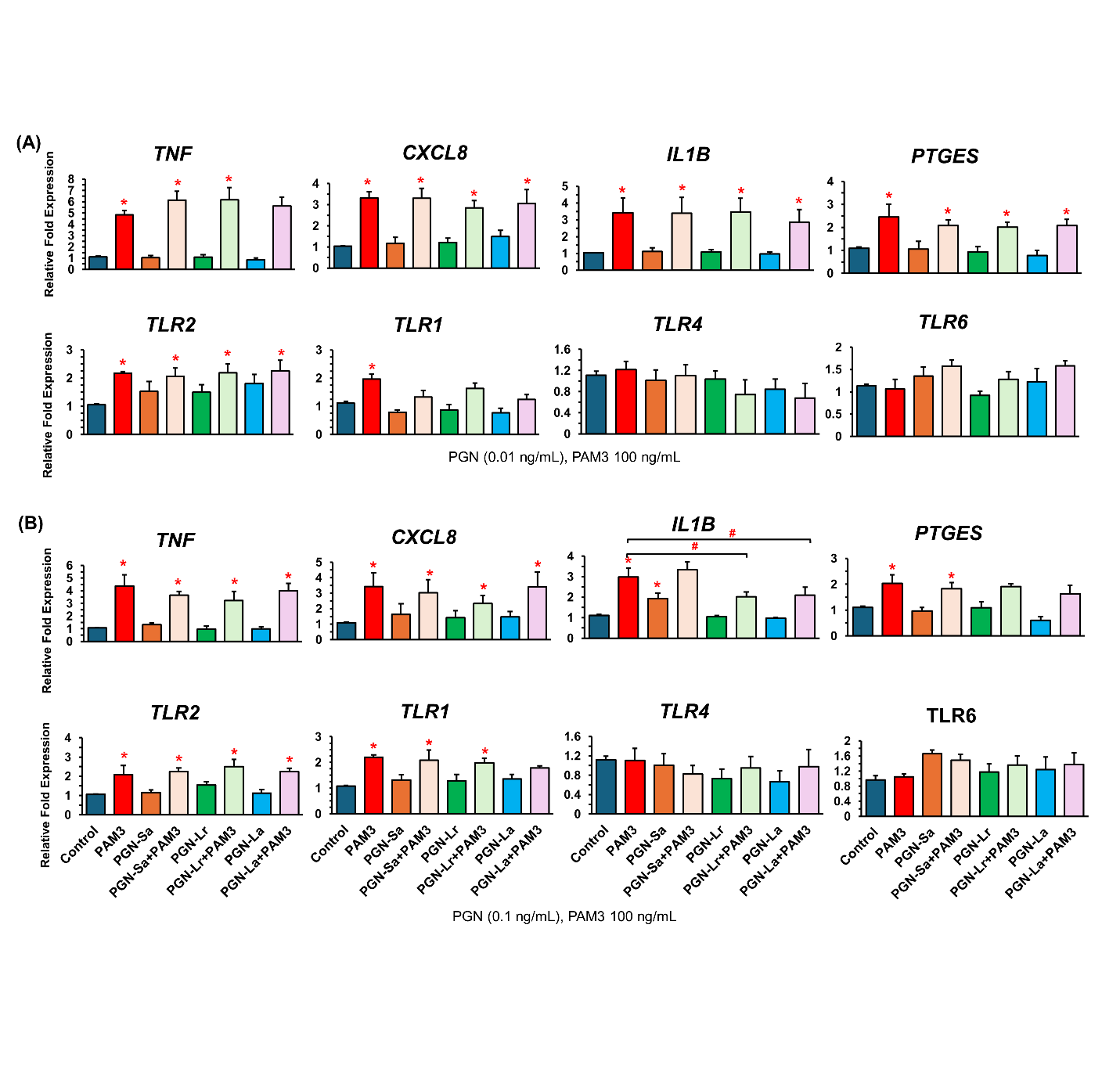
**

**Supplementary Figure 4. The impact of PGN-L on PAM3-induced inflammation in BEECs. (A)** PGN (Sa, Lr, La) pretreatment at (0.01 ng/mL), or **(B)** PGN (Sa, Lr, La) pretreatment at (0.1 ng/mL) for 24 h, then combination with PAM3 100 ng/mL for 3 h. Data are presented as the mean relative expression ± SEM from 5 independent experiments. Data were analyzed using the ANOVA with Bonferroni’s mean comparisons procedure. Asterisks show a significant difference compared with the control alone. (*P< 0.05, ** P<0.01, ***P< 0.001)


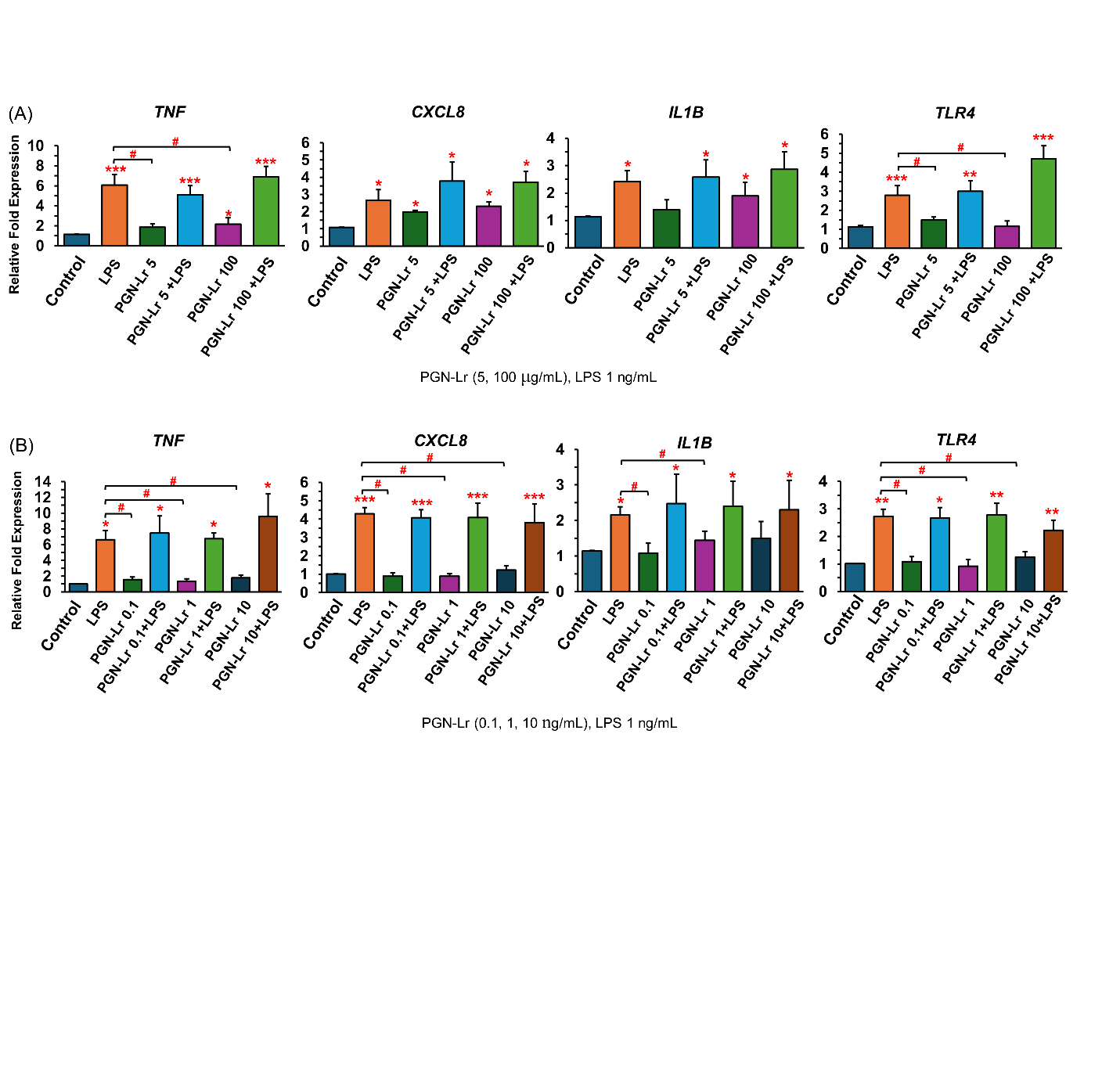


**Supplementary Figure 5. The impact of PGN-Lr on LPS-induced inflammation in BEECs. (A)** PGN-Lr pretreatment at (5, 100 μg/mL), or **(B)** PGN-Lr pretreatment at (0.1, 1, 10 ng/mL) for 24 h, then challenged with LPS 1 ng/mL for 1 h. Data are presented as the mean relative expression ± SEM from 3 independent experiments. Data were analyzed using the ANOVA with Bonferroni’s mean comparisons procedure. Asterisks show a significant difference compared with the control alone. (*P< 0.05, ** P<0.01, ***P< 0.001)

Supplementary Table 1. List of primers used in RT-qPCR

| **Gene** |  | **Primer** | **Sequence (5’–3’)** | **Accession no.** | **Product size (bp)** |
| --- | --- | --- | --- | --- | --- |
| ***ACTB*** |  | F | TCACCAACTGGGACGACATG | NM_173979.3 | 51 |
|  |  | R | CGTTGTAGAAGGTGTGGTGCC |  |  |
| ***TNF*** |  | F | CAAAAGCATGATCCGGGATG | NM_173966.3 | 51 |
|  |  | R | TTCTCGGAGAGCACCTCCTC |  |  |
| ***CXCL8*** |  | F | CCAATGGAAACGAGGTCTGC | NM_173925.2 | 51 |
|  |  | R | CCTTCTGCACCCACTTTTCCT |  |  |
| ***IL1B*** |  | F | AATCGAAGAAAGGCCCGTCT | NM_174093.1 | 51 |
|  |  | R | ATATCCTGGCCACCTCGAAA |  |  |
| ***PTGES*** |  | F | AAAATGTACGTGGTGGCCGT | NM_174443.2 | 51 |
|  |  | R | CTTCTTCCGCAGCCTCACTT |  |  |
| ***TLR1*** |  | F | ACCCTACTCTGAACCTCAAG | NM_001046504.1 | 142 |
|  |  | R | GACTGCACACTGGATTTCTG |  |  |
| ***TLR2*** |  | F | CATGGGTCTGGGCTGTCATC | NM_174197.2 | 51 |
|  |  | R | CCTGGTCAGAGGCTCCTTCC |  |  |
| ***TLR4*** |  | F | AGCCACGGCCATCCTCTCCT | NM_174198.6 | 740 |
|  |  | R | AGCTCAGGTCCAGCATCTTGGT |  |  |
| ***TLR6*** |  | F | CCTTGTTTTTCACCCAAATAGC | NM_001001159.1 | 154 |
|  |  | R | TAAGGTTGGTCCTCCAGTGAGT |  |  |

Supplementary Table 2. List of antibodies used for immunofluorescence (IF)

| **Target** | **Antibody** | **Source** | **Homology compared to bovine** | **Dilution** | **Working concentration (μg/mL)** |
| --- | --- | --- | --- | --- | --- |
| TNF | Mouse monoclonal anti bovine TNF primary antibody | BIO-RAD  (MCA2334) | 100% | 1:400 | 2.5 |
| TLR2 | Rabbit polyclonal anti human TLR2 primary antibody | Biorbyt (ORB11487) | 93% | 1:50 | 10 |
| PTGES | Rabbit polyclonal to Prostaglandin E Synthase/MPGES-1 | Abcam (ab62050) | 84.21% | 1:200 | 10 |
| IgG | Goat anti mouse IgG secondary antibody | Invitrogen  (A-11029) |  | 1:400 | 5 |
| IgG | Goat anti rabbit secondary antibody | Invitrogen  (A-11035 |  | 1:200 | 10 |
| NC | Mouse IgG2b negative control | BIO-RAD  (MCA691) |  | 1:40 (TNF) | 2.5 (TNF) |
| NC | Rabbit IgG negative control | Invitrogen  (AB_2532938) |  | 1:500 (TLR2) and 1:200 (PTGES) | 10 (TLR2) and 10 (PGES) |
